# Modelling of Immune Checkpoint Network Explains Synergistic Effects of Combined Immune Checkpoint Inhibitor Therapy and the Impact of Cytokines in Patient Response

**DOI:** 10.3390/cancers12123600

**Published:** 2020-12-02

**Authors:** Maria Kondratova, Emmanuel Barillot, Andrei Zinovyev, Laurence Calzone

**Affiliations:** 1 Institut Curie, PSL Research University, F-75005 Paris, France; emmanuel.barillot@curie.fr (E.B.); andrei.zinovyev@curie.fr (A.Z.); 2 INSERM, U900, F-75005 Paris, France; 3 CBIO-Centrefor Computational Biology, MINES ParisTech, PSL Research University, F-75006 Paris, France

**Keywords:** tumour immunology, immune checkpoints, immunotherapy, signalling network, logic modelling, intercellular communication, cancer systems biology, cell reprogramming, polarization, activation of T cells

## Abstract

**Simple Summary:**

The future of cancer immunotherapy relies on a combination of individually targeted therapies. However, a lot of experiments are needed to define the most effective combinations of drugs. A computational and modelling approach could help reduce the number of experiments and suggest optimal treatments to test. This article presents a logical model of T cell activation influenced by immune checkpoints, and explores the effect of these checkpoints, suggests mechanisms that would explain why some treatments might be better suited than others. The model includes not only programmed cell death protein 1 (PD1) and cytotoxic T-lymphocyte-associated protein 4 (CTL4) downstream pathways but also those of other immune checkpoints such as T cell immunoglobulin and ITIM (immunoreceptor tyrosine-based inhibition motif) domain (TIGIT), lymphocyte activation gene 3 (LAG3), T cell immunoglobulin and mucin domain-containing protein 3 (TIM3), cluster of differentiation 226 (CD226), inducible T-cell costimulator (ICOS), and tumour necrosis factor receptors (TNFRs).

**Abstract:**

After the success of the new generation of immune therapies, immune checkpoint receptors have become one important center of attention of molecular oncologists. The initial success and hopes of anti-programmed cell death protein 1 (anti-PD1) and anti-cytotoxic T-lymphocyte-associated protein 4 (anti-CTLA4) therapies have shown some limitations since a majority of patients have continued to show resistance. Other immune checkpoints have raised some interest and are under investigation, such as T cell immunoglobulin and ITIM (immunoreceptor tyrosine-based inhibition motif) domain (TIGIT), inducible T-cell costimulator (ICOS), and T cell immunoglobulin and mucin domain-containing protein 3 (TIM3), which appear as promising targets for immunotherapy. To explore their role and study possible synergetic effects of these different checkpoints, we have built a model of T cell receptor (TCR) regulation including not only PD1 and CTLA4, but also other well studied checkpoints (TIGIT, TIM3, lymphocyte activation gene 3 (LAG3), cluster of differentiation 226 (CD226), ICOS, and tumour necrosis factor receptors (TNFRs)) and simulated different aspects of T cell biology. Our model shows good correspondence with observations from available experimental studies of anti-PD1 and anti-CTLA4 therapies and suggest efficient combinations of immune checkpoint inhibitors (ICI). Among the possible candidates, TIGIT appears to be the most promising drug target in our model. The model predicts that signal transducer and activator of transcription 1 (STAT1)/STAT4-dependent pathways, activated by cytokines such as interleukin 12 (IL12) and interferon gamma (IFNG), could improve the effect of ICI therapy via upregulation of Tbet, suggesting that the effect of the cytokines related to STAT3/STAT1 activity is dependent on the balance between STAT1 and STAT3 downstream signalling.

## 1. Introduction

After the success of the new generation of immune therapies, referred to as immune checkpoint inhibitors (ICI), and which were awarded a Nobel Prize in 2018, immune checkpoint receptors have become an important center of attention in the search for efficient cancer therapies. However, the initial success and hopes of anti-programmed cell death protein 1 (anti-PD1) and anti-cytotoxic T-lymphocyte-associated protein 4 (anti-CTLA4) therapies showed some limitations. Although the checkpoint blockade treatments improved the therapeutic effect and opened a promising direction for future research [1,2], a majority of patients continue to show resistance, with a few exceptions such as Merkel cell carcinoma, a variant of melanoma (especially desmoplastic melanoma), MSI-high (microsatellite instability high) cancers, and Hodgkin’s lymphoma that respond to ICI therapy in ≥50% of cases [1,2]. Some published works focusing on other inhibiting and activating checkpoints, such as T cell immunoglobulin and ITIM (immunoreceptor tyrosine-based inhibition motif) domain (TIGIT), T cell immunoglobulin and mucin domain-containing protein 3 (TIM3), or inducible T-cell costimulator (ICOS), have already shown some interesting results [3,4]. A strong message that emerged from CTLA4 and PD1 studies is the understanding that the future of ICI lies in the combinatory therapy approach targeting different immune pathways, which proved to be more efficient than monotherapy [5]. Unfortunately, as of today, experimental data about the synergy between different checkpoints remain scattered and incomplete. We believe that a computational approach that facilitates the exploration of some of these combinations of targets could help decipher some complex mechanisms, prioritize the checkpoint candidates, and considerably reduce the time needed for experimental studies and validation.

Mathematical modelling has already proved to be useful in cancer analyses using nonlinear continuous approaches [6,7], and more and more models are being developed to understand the immune response and effect of therapies [8,9]. Logical formalism has been used in particular for its simplicity and its versatility, considering the limited available data that are used to build these models [10]. Some models focusing on signalling pathways altered in cancers [11,12,13,14], on processes related to the immune response [15,16], and on the effects of drug treatments [17,18,19] have already shown that a lot of insight can be gained with such a formalism. Some of these logical models have explored, in particular, the regulation of T cells in diverse contexts such as HIV [20], CD8+ exhaustion [9], and CD4+ activation [21]).

The methodology used in our study involves two consecutive tasks. The first one consists of recapitulating the knowledge dispersed in various sources into a unique network. For these purposes, we have built an influence network of T cell activation, including the most studied immune checkpoint inhibitors (PD1, CTLA4, TIGIT, lymphocyte activation gene 3 (LAG3), and TIM3) and activators (ICOS, cluster of differentiation 226 (CD226), and tumour necrosis factor receptors (TNFRs)). Very often, experimental publications focus on one gene, one checkpoint, or one signalling pathway, but very few offer a formal, comprehensive picture of a process. This is one goal of systems biology, to provide a holistic view of the systems of interest rather than studying their individual parts.

**Figure 1:**
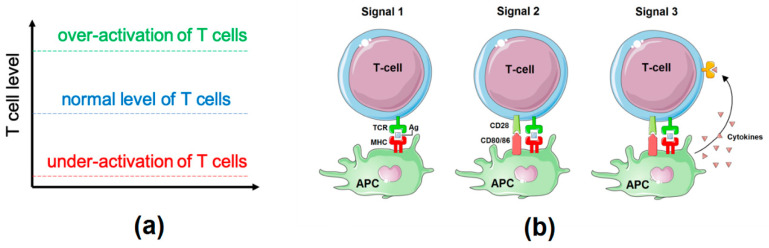
T cell activation. (**a**) Levels of T cell activity considered in the model: normal levels (blue) will be considered as no-activation; high levels (green) will be equivalent to a boost of T cell activation and referred to as T cell proliferation, and under-activation (red) corresponds to immunodeficiency cases. (**b**) Three steps of activation of T cells following adaptive immune response T cell activation: TCR activation (Signal 1), co-stimulation and co-inhibition of T cell receptor (TCR) signalling by immune checkpoints (Signal 2), modulation of T cell differentiation and effector functions by cytokines (Signal 3). APC: antigen-presenting cell; MHC: major histocompatibility complex.

Our model is based on the hypothesis discussed by Wei and colleagues [22], stating that: “Checkpoint blockade leads to an increase in positive costimulatory signals beyond levels that are achieved in normal scenarios. [...] enhanced efficacy could be derived from increased number of activated T cells and/or acquisition of new or enhanced functional properties due to super physiologic levels of costimulatory signalling”. In other words, when talking about T cell activation in the context of this study, it will mean, in fact, T cell activation above a physiological level (Figure 1a). For the rest of the study, we will consider that the “no activation status” for T cells corresponds to their normal physiological level (blue level in Figure 1a). Whether the T cell response to ICI relies mostly on the reactivation of pre-existing tumour-infiltrating lymphocytes or on the recruitment of novel T cells remains unclear [23,24]. However, our model could work in both contexts. Indeed, similar signalling pathways are involved in the primary activation of T cells and in their reprogramming or reactivation.

The second step translates the network into a mathematical model to interpret it dynamically. The natural translation of this influence network, referred to as an activity flow diagram in standard visualization language (Systems Biology Graphical Notation, SBGN) [25], into a mathematical model is done with the logical formalism (see Materials and Methods). The network then becomes a predictive tool that can be interrogated and modified to account for diverse cell conditions (environmental conditions, mutations, drug treatments, etc.). For instance, the model was designed to consider two types of T cell activation, based on their localization and their environmental conditions. The initial value of some variables of the model will define if the simulations take place in the lymph node (LN) and in the tumour microenvironment (TME). Finally, the model needs to be compared to published qualitative experimental results as a first validation, thus confirming that it can reproduce known facts about immune responses. In this study, we propose a framework that predicts the effect of therapies and suggests candidates for optimizing patient response to immune treatments.

## 2. Results

### 2.1. Influence Network: Proliferation, Survival, and Differentiation of T Cells Downstream of TCR Signalling

**Figure 2:**
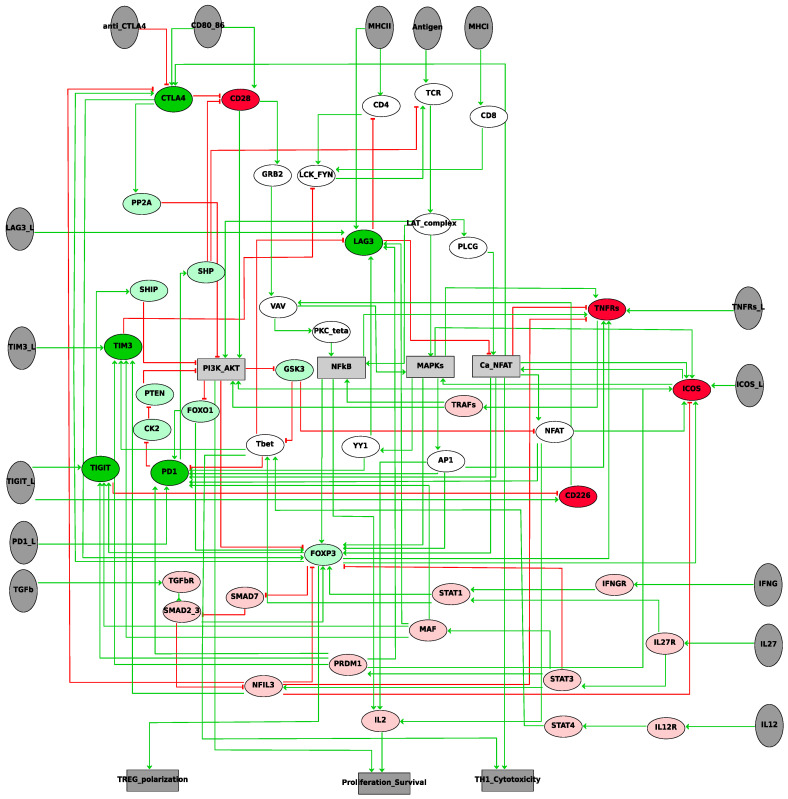
Influence network of TCR signalling. The antigen recognition by TCR leads to the formation and activation of LAT (linker of activated T cells) protein complexes, including, among other proteins, VAV and phospholipase C gamma (PLCG). LCK/FYN (p56lck/p59fyn) kinases also participate in this process via phosphorylations of key molecules of TCR complexes. This LAT-signalosome initiates several downstream signalling pathways such as Ca^2+^/nuclear factor of activated T-cells (NFAT), mitogen-activated protein kinases (MAPKs) (p38, c-Jun N-terminal kinase (JNK), and extracellular-signal-regulated kinase (ERK), and nuclear factor kappa-light-chain-enhancer of activated B cells (NFĸB) [27]. These pathways acting together induce the expression of interleukin-2 (IL2), which is a key regulator of T cell proliferation and clonal expansion. Another signalling pathway needed for a complete T cell activation is the phosphoinositide 3-kinase (PI3K)/AKT pathway, which reinforces the survival of activated T cells. Ligands for inhibiting and activating checkpoints as well as cytokines modulate the activity of the TCR signalling network on different levels. Key players of TCR signalling (white nodes) with activating (red nodes), inhibiting (green) checkpoints, and interactions with cytokines (pink nodes) are recapitulated into an influence network: nodes (ellipses) represent ligands (dark grey), proteins, genes or pathways, and edges account for positive (in green) or negative (in red) influence of one entity onto the others; square nodes show pathways (light grey) or phenotypes (dark grey).

Knowledge about the key players of the immune response to checkpoint therapies and how they interact is gathered and organized into a unique network (Figure 2). In this network, the full activation of T cells can be divided into three parts, following the process of the adaptive immune response (Figure 1b): (1) activation of T cell Receptors (TCR); (2) modulation of TCR downstream signalling by costimulatory and co-inhibitory immune-checkpoints; and (3) control of T cell activity by soluble agents (cytokines, prostaglandin, etc.).

The recognition of antigens by T cells requires a strong and stable cell-cell contact called the immunological synapse [26]. This contact leads to the formation of multiple intracellular molecular complexes, which include major histocompatibility complex (MHC)-bound antigen molecules from one side and TCR and CD4/CD8 receptors from the other side. CD4 recognizes the antigens of MHCII complexes and CD8 recognizes those of MHCI complexes. The antigen recognition by TCR leads to the formation and activation of TCR signalling network [27]. The main downstream players are Ca^2+^/nuclear factor of activated T-cells (NFAT), mitogen-activated protein kinases (MAPKs), nuclear factor kappa-light-chain-enhancer of activated B cells (NFĸB), and phosphoinositide 3-kinase (PI3K) pathways [27,28]. The activation of Ca^2+^/NFAT, MAPKs, and NFĸB pathways induces expression of interleukin 2 (IL2) and T cell proliferation. PI3K signalling is needed for the survival of activated lymphocytes (Figure 2). TCR alone could not maintain a stable downstream signal. Costimulatory receptors such as CD28 are needed for complete T cell activation, whereas co-inhibitory receptors prevent hyperactivation of immune cells and provide attenuation of the immune response. This study focuses on checkpoints with known molecular mechanisms of action confirmed by many experimental studies. The network includes five inhibiting immune checkpoints, PD1, CTLA4, TIGIT, LAG3, and TIM3, and three activating immune checkpoints, ICOS, CD226, and TNFRs.

The network focuses on two cell fates: the first one is the proliferation downstream of IL2 and PI3K (*Proliferation_Survival* phenotype), and the second one is the T cell differentiation [29] modulated by cytokines and specific transcription factors (*Th1_CTL* and *Treg*). Currently, immunologists define about ten basic groups of effector T cells and a lot of subgroups [30]. Among these ten groups, two or three might be of particular interest in the context of cancer biology [30]: the regulatory T cell phenotype driven by FOXP3 and partially FOXO1 (referred to as *Treg* in the model), and Th1 (CD4+) or cytotoxic (mostly CD8+ but also CD4+) phenotypes driven by Tbet (T-box transcription factor TBX21) (referred to as *Th1_CTL* in the model).

The T regulatory (Treg) cell type is defined in the literature as the population of CD4 positive (CD4+) cells that stably express a high level of FOXP3 [31]. Regulatory T cells play an immunosuppressive role in the body: they express a high level of inhibiting immune checkpoints on their surface and produce immunosuppressive cytokines TGFβ and IL10. FOXP3 and FOXO1 transcription factors play a critical role in the regulation of these immunosuppressive phenotypes. The Treg population consists of two subpopulations of natural Tregs (nTregs) and inducible Tregs (iTregs). The first subpopulation will not be described here because nTregs represent a very specific group of T cells, which develop in the thymus and whose differentiation does not depend on the mechanisms of T cell activation explained above, whereas iTreg cells develop from mature CD4+ conventional T cells, and could be influenced by both immune checkpoints and cytokine signalling [31]. For the rest of the study, iTregs will be referred to as Tregs. Treg phenotype was first described for CD4+ cells only. Later, it was shown that CD8+, as well as CD4+, could express FOXP3 in certain conditions [32,33]. Although the total number of CD8+ FOXP3+ cells is small, they could play an important role in the tumour microenvironment (TME). Both CD4+ FOXP3+ and CD8+ FOXP3+ cells inhibit tumour rejection and promote tumour growth [34]. As a general observation, a high level of FOXP3+ cells in the TME is associated with a bad outcome [35,36]. FOXO1, another key transcription factor of Treg development, is especially important for the differentiation of iTregs [37,38].

The third transcription factor, Tbet, is known to regulate the development of Th1 cells (CD4+) and cytotoxic CD8+ cells (together with Ca/NFAT signalling). Among the T cell population, both cytotoxic (CTLs) and Th1 cells have a significant impact on tumour rejection and patients’ survival. CTLs directly recognize tumour cells and kill them via lytic granule exocytosis, and apoptosis induction and Th1 cells interact with antigen-presenting cells (mostly macrophages) and maintain the activity of innate immune response against tumours [39]. The two T cell populations produce a lot of inflammatory cytokines, such as interferon gamma (IFNG). The production of inflammatory cytokines by Th1 and CTLs and the expression of cytotoxic agents in CTLs both require Tbet activity. For simplicity reasons, we chose to ignore these feedbacks in this version of the model and study the ON and OFF effects of the cytokines, how their signals integrate to the immune checkpoints, and their impact on the cell fate. That way, we can focus on the effect of the tumour microenvironment on T cell activation.

The expression of these factors is regulated by the crosstalk between the TCR signalling pathway, immune-checkpoints, and cytokines. Thus, in the T cell activation network, they play the role of molecular “hubs” integrating all three types of signals (Figure 2). The main cytokine pathway upregulating FOXP3 expression is TGFβ/SMAD signalling [40,41]. IL12 and IFNG upregulate the expression of Tbet via signal transducer and activator of transcription 4 (STAT4) and STAT1 [42], IL27 and IFNG upregulate the expression of both Tbet and FOXP3 via STAT1 [43,44], but IL27 can also inhibit FOXP3 expression via STAT3 [45]. The final network contains a number of feedback loops, including these transcription factors that potentially contribute to the regulation of the expression and the functional activity of the immune checkpoints. PI3K pathway, downstream of TCR receptors, upregulates the expression of Tbet through the glycogen synthase kinase-3 (GSK-3), which leads to the inhibition of PD1 expression [46,47]. PI3K pathway blocks the transcription factor FOXP3 expression [48], which regulates the expression of many checkpoints such as CTLA4, ICOS, and TNFRSF18 (GIRT) [49]. Note that CTLA4 is also able to upregulate FOXP3 expression [49,50], thus forming a positive feedback loop between these two proteins.

Activation of the PI3K pathway leads to the phosphorylation of FOXO1, causing its exclusion from the nucleus. TIGIT stimulation represses the PI3K/AKT pathway via SHIP-1, resulting in the activation of FOXO1 [51]. FOXO1 is one of the transcription factors promoting the expression of PD1 [52].

The phenotypic outputs, or read-outs, of the model, can be understood as possible fates of the cell. The three outputs that are explicitly modeled here are: *Proliferation_Survival*, which can be understood as T cell proliferation and the number of T cells that could be attracted to the tumour, *Th1_CTL*, which corresponds to the differentiation into the Th1 cell types, and *Treg*, which corresponds to the differentiation into the Treg cell types.

The network is generic and will be made specific to different cell types (CD4+ or CD8+), cell conditions (treated or non-treated), and localization (in the lymph node or in the tumour microenvironment) based on specific sets of initial conditions (Table 1).

As previously mentioned, the described interactions are recapitulated into a single network (Activity Flow in the standard format SBGN [25]) drawn with the software GINsim [53] (Figure 2). The key genes that are found in this network (Figure 2) were selected based on their importance in the TCR signalling and their interaction with the immune checkpoints. The picture is not comprehensive but contains the major genes that may be representative of longer chains of events or of pathways. The details of these interactions and their references can be found in Supplementary Material Table S1.

### 2.2. Logical Model of TCR Network

The network of Figure 2 is static: it shows the possible influences of one entity onto the others, but the interpretation is limited. We propose to add a dynamic layer on top of these networks by considering that each of these nodes can take two values, 0 or 1, and by assigning to each of them a logical rule. These rules will define the condition for a node to change its value from 0 to 1—and vice versa—by combining the influence of the input nodes with logical operators OR (|), AND (&), and NOT (!). For instance, TCR will be set to 1 in the presence of the antigens and of the complex formed by LCK and FYN, and in the absence of SHP (TCR = Antigens & LCK_FYN & !SHP). The activation of some components of the network depends on the presence of some ligands (dark grey ellipses in Figure 2). If the ligands are absent, the downstream events will never be activated.

The model is first constructed in GINsim [53] and then exported in MaBoSS format [54] to simulate the model stochastically and provide a semi-quantitative interpretation of the results. The model in GINsim and MaBoSS formats can be found in the Supplementary Material and in the GINsim repository (see Materials and Methods for more information about the model formats).

### 2.3. Model Validation. Simulations of Different Aspects of T Cell Biology

To validate the model coherence, we confirmed with some in silico experiments (model simulations) that it was able to reproduce reported behaviors such as T cell proliferation without any inhibitory immune checkpoints or the gradual reduction of this proliferation potential in the presence of some checkpoints, etc.

T cell regulation raises a lot of questions that could be tackled by mathematical modelling: How to compare the impact of different checkpoint receptors in TCR signalling inhibition? What is the difference between CD8+ and CD4+ T cell response to immunotherapy? etc. The activation of the different model input combinations can reproduce a variety of environmental and contextual cell conditions that are detailed in Table 1 (e.g., localization and cell types).

In the first condition simulated by the model (Table 1), we explore the impact of individual checkpoint receptors in the modulation of TCR signalling. Each of these checkpoints targets different downstream pathways, and their individual activation may provide some hints on the power of their inhibition. Because of the crosstalk between these pathways, it is difficult to intuitively anticipate the contribution of individual checkpoints.

For the second cell condition, we have separated the two events that occur in the lymph node (LN) and in the tumour microenvironment (TME). The first step (so-called priming) happens in the lymph node where a naive T cell meets a dendritic cell presenting tumour antigens with a rather good affinity to TCR receptor expressed on the surface of the T cell. This stage leads to the proliferation of activated cells, also called clonal expansion because all descendants express the same type of TCR receptors as the first activated T cells [55]. Then, these T-lymphocytes start to differentiate and migrate from the lymph node to the infected peripheral tissues or tumour, where the second (effector) step of T cell activation takes place. When differentiated T cells meet the antigen again and recognize it, the response can lead to direct killing of infected or transformed cells in the case of cytotoxic CD8+ cells, to the expression of inflammatory cytokines in the case of Th1 cells, and to the production of immunosuppressive molecules in the case of inducible Tregs [55]. Naive and early activated T cells in LN and effector T cells in TME express different combinations of checkpoint receptors. The modulation of the checkpoint signalling by cytokines play a key role in the process.

Finally, the last condition focuses on CD8+ and CD4+ activation. MHCI complexes activate CD8+ cells, and MHCII complexes are recognized by CD4+ cells. If the priming occurring in the LN is almost the same for CD4+ and CD8+ cells, the fate differs for the secondary activation in the TME; TCR activation of effector CD4+ cells is CD28-dependent, whereas the cytotoxic activation of CD8+ cells does not need CD28 co-stimulation, because non-immune cells normally do not express CD80/CD86 molecules. We show the synergistic effect of combined immune checkpoint treatments in the context of CD8+ and CD4+ T cells and predict the optimal combination for an efficient patient response.

We detail below each of the model simulations of specific cell conditions. All results can be reproduced with the Jupyter notebook provided in the GitHub repository (*Supp_mat_jupyter_notebook_model_analysis* in https://github.com/sysbio-curie/ICI).

### 2.4. Role of Individual Inhibiting Immune Checkpoint Receptors in TCR Signalling Modulation

**Figure 3:**
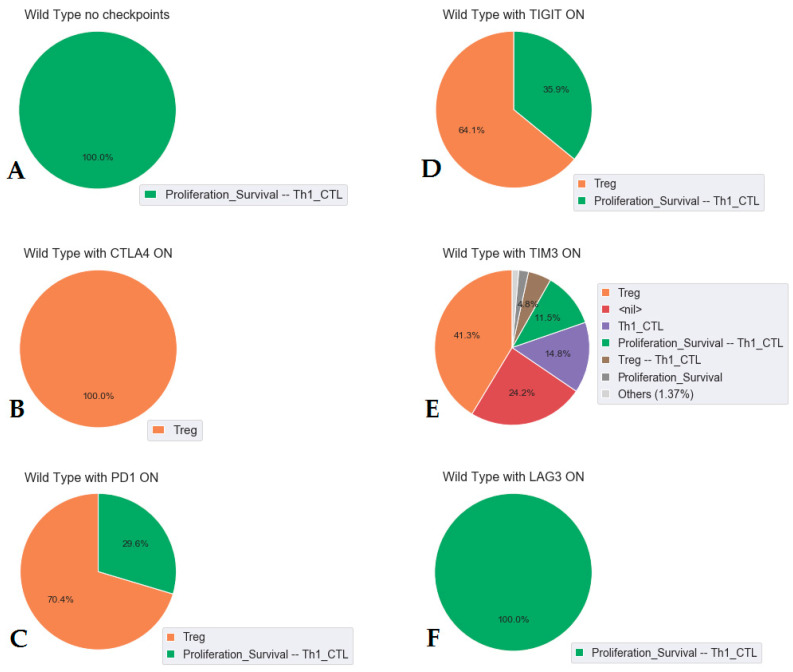
Impact of the presence of individual immune checkpoints on the fate of T cells. The model is simulated with no immune checkpoint (**A**), with only CTLA4 ligands ON (**B**), with only PD1 ligands ON (**C**), with only TIGIT ligands ON (**D**), with only TIM3 ligands ON (**E**), and with only LAG3 ligands ON (**F**). In the legend, the presence of a phenotype means that it corresponds to the asymptotic solution of the simulations in all the conditions (from **B**–**F**). If Treg is mentioned, it should be understood as: *Treg* is ON, but *Proliferation_Survival* and *Th1_CTL* are OFF.

We tested the impact of each of the inhibitory immune checkpoints (CTLA4, PD1, TIM3, LAG3, and TIGIT) by activating their respective ligand one by one, and reported on the cell capability to proliferate (activation of the phenotype *Proliferation_Survival*), and the proportion of Th1 (*Th1_CTL*) and Treg (*Treg*) cell populations (Figure 3). Figure 3 shows simulations for a generic T cell (CD4+ or CD8+ cells).

In our model, the activation of the CTLA4 pathway completely inhibits both *Proliferation_Survival* and *Th1_CTL* phenotypes and maintains *Treg* phenotype (Figure 3B). PD1 and TIGIT are not as strong as an inhibitor as CTLA4 (70% and 65%, respectively, Figure 3C,D). TIM3 shows a drastic reduction of the Treg population (around 50%, Figure 3E), but because of its downstream influence on TCR (through the indirect effect of LCK_FYN), it reduces proliferation (Figure 2). LAG3 ligands alone do not demonstrate any inhibiting activity because their expression is not stable in the model as a result of Tbet activation. Only when LAG3 is overexpressed (its activity was forced to 1), is the inhibiting effect observed (Figure 3F and Jupyter notebook).

These results correspond to our current knowledge about immune checkpoint biology. The general consensus of the immunological community based on many experiments is that CTLA4 is the strongest inhibiting checkpoint, PD1 is weaker than CTLA4, and TIGIT, LAG3, and TIM3 are considered even weaker checkpoints [3]. It is in good agreement with the results of our simulations, except for TIGIT, which our model points out as a rather strong checkpoint.

TIGIT (T cell immunoglobulin and ITIM domain) is a member of the CD28 family. It binds to two ligands, CD155 and CD112, which are expressed on APCs, T cells, and a variety of non-hematopoietic cell types, including tumour cells. It has been reported that TIGIT interacts with much higher affinity with CD155 than with CD112. CD226 (DNAM-1) binds to the same ligands and, together with TIGIT, forms a complex in which CD226 delivers a positive costimulatory signal, whereas TIGIT triggers inhibitory signals [3,56]. Engagement of TIGIT through CD155 induces the recruitment of SHIP1 (SH2 domain-containing inositol-5-phosphatase 1), which in turn blocks signal transduction through the PI3K pathway [51]. Activation of the PI3K/AKT pathway is a key step in T cell activation. The significant value of inhibiting CTLA4 and PD1 could be explained by their negative influence on PI3K/AKT pathway, and our model assumes that TIGIT could have a stronger inhibiting potential than that of TIM3 or LAG3 (which are not directly involved in PI3K regulation).

In our model, the CTLA4 signal is so strong that it dominates over all the other inhibitors. If cells express both CTLA4 and PD1, PD1 blockade alone is not able to reactivate them. In reality, the domination of CTLA4 is probably not complete; the PD1 blockade alone is able to enhance the proliferation of both PD1+ CTLA-4− and PD1+ CTLA-4+ T-lymphocytes, although it is unable to completely restore the proliferation of PD1+ CTLA-4+ cells (Table 2, and Jupyter notebook on comparison of model simulations with published experimental results *Supp_mat_jupyter_notebook_Exp_validation* available at https://github.com/sysbio-curie/ICI) [57].

### 2.5. Two-Step Simulations of T Cell Response in Cancer Treated with PD1 and CTLA4 Inhibitors

The steps leading to the T cell response can be illustrated by at least two spatially and temporally separated events that occur first in the lymph node (LN) and then in the tumour microenvironment (TME). Throughout this process, several naive T cells primed in the LN give rise to millions of differentiated T cells in the TME; many of these cells are subtypes of CD4+ cells (Th1, Th2, Th17, Treg, etc.), each of which plays a particular role in the anti-tumour immune response. The specific immune response first necessitates T cell proliferation, i.e., clonal expansion, in the LN, followed by migration of these T cells to the tumour bed, which triggers a specific response. The presence or absence of the immune checkpoints in these environments affects the anti-tumour immune response. Note that it has been reported that there exist different repertoires of immune checkpoint receptors in both the LN and the TME [58,59].

To reproduce these complex and dynamical cell conditions with a relatively simple logical model, we had to make several assumptions. First, T cell response is simulated as a two-step process, where they are described as independent and isolated processes. The two stages have different initial conditions that mimic their respective context. These initial conditions correspond to the presence or absence of ligands or known and reported activity of some genes or proteins in the literature. Second, the biological interpretation of the model outcomes is the combined result of the two conditions. For instance, if the priming of T cells is not efficient in the first phase, the response in the TME will not be efficient either. Third, the balance between anti-tumour and pro-tumour immune responses is described as a ratio between *Th1_CTL* and *Treg* phenotypes. For simplicity, the impact of other T cell subtypes is considered less critical and is ignored, as well as the impact of innate immunity. Fourth, in the first stage, the stage of clonal expansion, cells express CTLA4, ICOS, and TNFRs only. In this context, T cells can proliferate and then start to differentiate into specific subsets. Finally, in the second stage, the stage of differentiation and effector function in the TME, T cells express all checkpoints except CTLA4. They continue to proliferate, differentiate, and acquire their effector functions. Note that in reality, a minor population of tumour-infiltrating T cells continue to express CTLA4 alone or in combination with other checkpoints, but most of the published results agree that the main effect of CTLA4 takes place in the LNs [22,60].

The impact of the presence of these checkpoints in the LNs and TME was explored for both CD4+ and CD8+ cells (Table 1). Details are provided in the Jupyter notebook of the model simulations at the following address: https://github.com/sysbio-curie/ICI.

### 2.6. Comparison of the Model Simulations with Experimental Observations

The treatments in the different environments, LNs and TME, will not have the same effects. The expected results are recapitulated in Table 3 and are confirmed by experimental results (last column).

Our model is able to predict and to explain the differences between the effect of monotherapy and of the combined ICI therapy (anti-PD1 and anti-CTLA4). However, some discrepancies persist between the anti-PD1 and anti-CTLA4 monotherapies: about 30% of the patients with anti-PD1 treatments show good clinical outcomes vs. 10%–15% of patients with anti-CTLA4 treatments [5]. We confirm with the model that the anti-CTLA4 treatments allow clonal expansion in the lymph nodes, but this is not enough for an efficient immune response, because differentiation into specific T effectors is blocked by immune checkpoints in the tumour bed. Thus, a second treatment involving PD1 inhibition favors the production of Th1 cells.

It is important to note that there are some factors that are not stimulated by the model and that have an impact on the efficacy of the treatments, among them the age of the patients, the tumour size and its localization, pharmacological properties of different antibodies that are used, etc.

We compared further the results of the logical model with experimental data of different anti-CTLA4 and anti-PD1 reported treatments in mice models [57] (Table 2). We focused our comparison on the three phenotypes of the model: T cell proliferation *(Proliferation_Survival)*, *Th1_CTL* proportion, and *Treg* proportion. The simulations of the logical model show good qualitative correspondence with experimental observations. However, more human data would be needed to validate our model and truly translate our predictions to possible treatment options.

### 2.7. Role of Cytokines in the Modulation of the Effect of the Checkpoint Therapy

The key transcription factors that are regulated downstream of the immune checkpoints are also targeted by the cytokine pathways. In this study, we focused on three major transcription factors: FOXP3, FOXO1, and Tbet. The activation of cytokines, such as IFNG and IL12, upregulates the expression of Tbet via STAT1 and STAT4 pathways, respectively [61,62]. The immunosuppressive cytokine, TGFβ, upregulates FOXP3 expression via Smads [40,41]. High levels of IL12 and IFNG are usually associated with increased activity of Th1 cells and cytotoxic lymphocytes, whereas the TGFβ pathway is a major regulator of Treg cells.

Some cytokines have dual roles. In certain conditions, IFNG can also upregulate FOXP3 expression via STAT1 [44]. Similarly, IL27 is able to activate STAT3 and STAT1 pathways [43]. FOXP3 can then simultaneously be activated downstream of IL27/STAT1 axis [44] and downregulated downstream of IL27/STAT3 directly or via NFIL3 [45,49]. IL27 induces the expression of many inhibiting checkpoints via the transcription factors MAF and PDM1 [63]. The combination of these different effects on one pathway highlights the complexity around the regulation of the immune response and shows the importance of the contextual conditions for a proper response.

**Figure 4:**
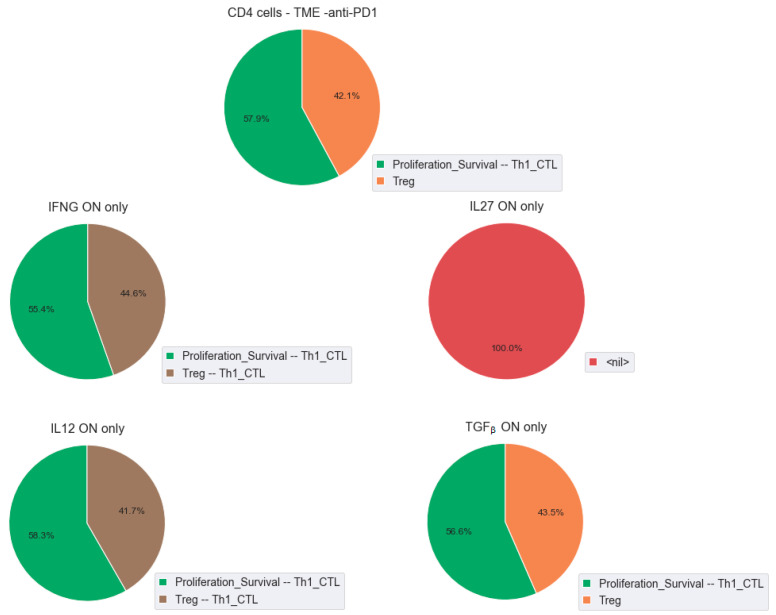
Effect of cytokines on CD4+ cells. CD4+ cells: in the absence of the cytokines (top panel), in the presence of IFNG only (middle left panel), IL12 only (bottom left panel), IL27 only (middle right panel), TGFβ only (bottom left panel). In the legend, the presence of a phenotype means that it corresponds to the asymptotic solution of the simulations in all the conditions. If Treg is mentioned, it should be understood as: *Treg* is ON, but *Proliferation_Survival* and *Th1_CTL* are OFF.

To study the influence of the cytokines and the transcription factors in T cell activation, we simulated the CD4+ cell conditions and compared the effect of anti-PD1 therapy in the presence of the cytokines. We first tested the effect of the individual cytokines: IFNG, IL12, IL27, and TGFβ. We observed that IFNG and IL12 increased the ratio Th1/Treg with a mixed phenotype, Treg-Th1, whereas TGFβ did not show any change in phenotype proportions. IL27 has an interesting effect: acting alone, it blocks both Th1 and Treg phenotypes (Figure 4). It confirms experimental observations that the effect of this cytokine in cancer is very context-dependent [64].

Since the targets of the cytokines are the transcription factors, we confirmed that the cell behaviours were equivalent to forcing the activity of Tbet for IFNG and IL12. However, forcing the activity of FOXO1 or FOXP3 did not reproduce the presence of the cytokines IL27 and TGFβ, but we showed that in these cases, the cell fate is STAT3-dependent and not STAT-1-dependent (Supplementary Figure S1).

The role of the cytokines has been explored in several studies, and our model confirms their role in an efficient response to treatment. In a study focusing on the differences between responders and non-responders to an anti-PD1 therapy [65], we can confirm that upregulation of IFNG and IL12 signalling pathways in responders could compensate for the presence of other inhibiting checkpoints. Indeed, non-responders and responders have a surprisingly high level of several inhibiting checkpoints (PD1, TIM3, TIGIT, and LAG3, and PD1, TIGIT, and LAG3, respectively), which could, potentially, alter the effect of the anti-PD1 therapy. However, the presence of Tbet downstream of IFNG and IL12 in responders seems to set appropriate conditions for an optimal response (Supplementary Table S2 and Supplementary Figure S2).

## 3. Discussion

The role of inhibiting immune checkpoint and their interplay in the activation of T cells reveals a complex network of interactions that highlights how unpredictable the response to treatments is. As a first step towards a better understanding, we have constructed a model to explain and predict the effect of different types of combinatory immune-checkpoint inhibitor therapy. The model that we have developed has allowed the formalization of some hand-waving explanations of the interplay between the inhibitory immune checkpoints. Our model shows that in the case of co-expression of CTLA4 and PD1 in the same cell, anti-PD1 therapy will not be efficient because CTLA4 is the strongest inhibiting checkpoint receptor, which corresponds to observations reported in mouse cells [57] (Table 2). We have also shown that the two-step process may explain the efficiency of the combined anti-CTLA4 anti-PD1 therapies and have suggested a potential efficient effect of anti-TIGIT therapies.

The formalism we are using is coarse grain and does not allow quantitative predictions, but we anticipate that some effects may be modulated by the strength of the inhibitions of each of the present immune checkpoints and that combinations of partial inhibitions of the inhibitory checkpoints may help reduce toxicities. Moreover, the balance between activating and inhibiting checkpoints needs to be explored further with quantitative studies. Which ratio between CTLA4 and CD28 on the T cell surface could provide an efficient blockade of T cell coactivation, 1:1, 1:2, 1:100? How much IFNG signalling would be needed to compensate for the immunosuppressive effect of TGFβ or IL10? The model can point out some experiments that would answer these questions. We will explore these effects in future prospects when quantitative published data are available.

What we have been able to do with this Boolean model is to explain formally in simple terms with a rather simplified, yet complex, network the possible interactions at play in the efficiency of ICI therapies. Combining this model with more complex complementary models might be more insightful to answer other types of questions. Two recent models have been published on immune checkpoint therapies [8,9]. If these models show a good overlap with our model, they focus on different aspects: Hernandez et al. provide a very detailed model (up to 200 nodes) of the downstream effect of CTLA4 and PD1 and focus on the methodology to explore such complex models, whereas Bolouri et al. developed a model exploring the steps involved in CD8+ T cell exhaustion and the impact on the efficacy of the treatments. Analyzing these different models separately and combining them into a comprehensive model would greatly improve our understanding of the immune checkpoint therapies. However, it is important to keep in mind that these models were built for different purposes, and combining dynamical models is a challenge of its own. We also believe that simple models allow to keep the reasoning straightforward and facilitate the communication between biologists and modellers, the purpose being to fill the gap between theory and practice in immunological studies. The complexity and detailed description of these models should then be incremental.

The integration of disseminated articles about immune checkpoint regulation into a single network has highlighted some discrepancies, gaps, and points of interest. First, there are a number of articles that have shown that the inhibition of certain immune checkpoint receptors could result in upregulation of others. This effect looks like a natural compensatory mechanism of the immune system to protect our body from overactivation of immune activity. However, this phenomenon could have a great negative impact on the efficiency of ICI therapy. Little is currently known about the molecular mechanisms, and as new interactions are unveiled, they may affect the network predictability. One example of the model limitations due to lack of knowledge is our simulation of responders and non-responders of anti-PD1 therapies [65]: our model correctly explains and predicts the effect of cytokines on anti-PD1 therapy response, but it could not predict the upregulation of TIGIT and LAG3 expression after the anti-PD1 treatment.

The logical rules associated with each node of the network may be crucial in the dynamics of T cell activation in different contexts described in this study. For instance, when node A receives both a positive and a negative signal from genes B and C, respectively, there are several options: A can be activated when B is present and when C is absent (B AND NOT C) or when B is present or C is absent (B OR NOT C). In the second case, there will be situations when A will be activated even in the presence of its inhibitor C. For our model, we have chosen the “inhibitory bias”, which creates extreme situations, whereas, in reality, the inhibition could be partial. In the case of inhibitory immune checkpoints, experimental data partially confirm that immuno-suppressive signals are stronger than immune-activating signals. Indeed, for complete activation of immune cells, several synergistic signals might be observed: TLR and cytokines for innate immune cells [66], TCR costimulation and cytokines for T cells [22], and only one inhibiting agent (either a checkpoint or a cytokine) could block the process. This Boolean model does not pretend to provide quantitative predictions but rather explore some qualitative features that would suggest the role of the immune checkpoints in different contexts and help search for new combinations of ICI drugs.

## 4. Materials and Methods

### 4.1. Construction of the Network of the Immune Checkpoint Inhibitor Response

The network was built from disseminated published facts and integrated into a comprehensive network of interactions. The network is based on the SBGN Activity Flow format. The network was created using the methodology developed before [67,68]. Molecular interactions reported in the scientific articles were manually curated, and the information extracted from the papers was used for reconstruction and annotation of the maps. Two types of articles were used for map annotation: (i) experimental innate-immunity-specific articles directly or indirectly confirming molecular interactions based on mammalian experimental data; (ii) review articles.

The chemical reactions involving the key players of the processes and reported in the publications were interpreted and translated into influences, either positive or negative. Each node represents a molecular entity, which englobes ligands, receptors, (phosphorylated or non-phosphorylated) proteins, cytokines, etc. Each arrow represents an influence, positive or negative, interpreted from the biochemical reaction it is representing: a complexation, a (de)phosphorylation, a degradation, etc.

All publications used to build the network are gathered into a Supplementary Document, *Supp_Mat.doc*, where each interaction is supported by at least one publication.

### 4.2. Logical Modelling and Simulations

The network was then translated into a mathematical model. To each node of the network, a logical rule is formulated linking the input of a node with the logical connectors AND, OR, and NOT.

The solutions of the Boolean model are called attractors. They can correspond to stable states or limit cycles. A stable state corresponded to a state whose successor is itself, whereas a limit cycle corresponded to a series of states that repeat themselves in a recurrent order.

The attractors of the Boolean model could be computed exactly with GINsim [53] or estimated with a stochastic approach with MaBoSS [54]. Because of the high number of inputs, there are many stable states for each combination of inputs. For this reason, we focused our analysis on the stochastic simulations of the model to provide semi-quantitative results.

MaBoSS uses Markov chains on the Boolean network and computes time trajectories of the probabilities of the model variables and of the individual molecular entities. With a set of initial conditions and transition rates for each of the variables (speed of activation and of inhibition, set here to standard value 1), MaBoSS simulated a high number of trajectories that are then averaged to provide a single trajectory over time. The solutions are represented as pie charts, which are equivalent to asymptotic solutions of the model states. They correspond to the probabilities to reach each of the phenotypes of interest, i.e., the proportion of the trajectories with the corresponding phenotype equal to 1 at the end of the simulation. The time trajectories are available in the Jupyter notebooks: they can be interesting for the case of transient behaviours.

### 4.3. Model Simulation of Immune Checkpoint Inhibiting Treatments

The immune checkpoint inhibitor treatments were simulated by forcing the immune checkpoint receptor activity to zero. In the case of anti-CTLA4, we also added a node “anti-CTLA4” to explore the possibility to partially inhibit the receptor activity and measure the impact of this partial inhibition on TCR signalling. The results are presented in the Jupyter notebook but not discussed here.

### 4.4. Model Accessibility and Data Availability

All results presented in this study were gathered in Jupyter notebooks to ensure reproducibility and allow the simulations of any in silico experiments or cell conditions. They are accessible at the address: https://github.com/sysbio-curie/ICI.

Upon publication, the model will be deposited in public databases (GINsim repository: http://ginsim.org/models_repository in zginml and MaBoSS formats, and in BioModels database in SBML format) for its wide distribution and its reuse.

### 4.5. Data Used for Experimental Validation

Supplementary Table S1 recapitulates the data extracted from the initial publication (Gide et al., 2019) and that focused only on the genes of interest for our study. The work explored the patient response to anti-PD1 treatments, and gene expression was reported for responders vs. non-responders.

## 5. Conclusions

Our model illustrates the main pathways that are activated or inactivated in response to the checkpoint inhibitors. The model is able to mimic the effect of treatments and suggests important roles of other checkpoints, such as TIGIT. Moreover, the model predicts that STAT1 and STAT4-related cytokines such as IL12 and IFNG could improve the effect of ICI therapy via upregulation of Tbet, suggesting that the effect of the STAT3–STAT1 are dependent on the balance between STAT1 and STAT3 signalling.

## Figures and Tables

**Table 1:** Definition of cell conditions: the nodes of the model listed in this column inform on which of these components are initially active (set to 1), and these initial conditions correspond to cell environmental conditions, cell localization, or cell types. PD1: programmed cell death protein 1; LAG3: lymphocyte activation gene 3; CTLA4: cytotoxic T-lymphocyte-associated protein 4; TIM3: T cell immunoglobulin and mucin domain-containing protein 3; TIGIT: T cell immunoglobulin and ITIM (immunoreceptor tyrosine-based inhibition motif) domain; CD226: cluster of differentiation 226; ICOS: inducible T-cell costimulatory; TNFRs: tumour necrosis factor receptors.

Cell Conditions	Initial Conditions
1. Role of individual inhibiting immune checkpoint receptors in TCR signalling modulation	CD80/86 (ligand for CTLA4 or CD28) only
	PD1_L (ligand for PD1) only
	LAG3_L (ligand for LAG3) only
	TIM3_L (ligand for TIM3) only
	TIGIT_L (ligand for TIGIT or CD226) only
2. Priming in lymph node (LN) vs. secondary tumour microenvironment (TME) activation (two-step process)	LN (immune checkpoints active): CTLA4, ICOS, TNFRs
	TME (immune checkpoints active): ICOS, TNFRs, PD1, TIGIT, LAG3, TIM3
3. CD8+ vs. CD4+ cells	CD8+ (ligands): MHCI
	CD4+ (ligands): MHCII

**Table 2:** Experimental results of anti-CTLA and anti-PD1 treatments in CD8+ cells (↑ corresponds to upregulation and ↓ corresponds to downregulation of phenotype after ICI treatment. Note that we refer to the intensity of the response as follows: ↑↑↑>↑↑>↑, ↓↓↓<↓↓<↓) [57].

Cell Population	Type of ICI Therapy	T Cell Proliferation		Th1_Cytotoxicity		Treg	
		Model	Experimental Data (Different Methods)	Model	Experimental Data IFNG/pTbet/GZMB (Granzyme B)	Model	Experimental Data ** Treg Ratio/IL10/TGFβ
All TILs	anti-CTLA4		NA	↑	↑/NA/NA	↓	No effect/No effect/- -
All TILs	anti-PD1 (anti PD1-L)		NA	↑	↑/NA/NA	↓	↓/↓/↓
All TILs	anti-CTLA4 & anti-PD1		NA	↑↑	↑↑/NA/NA	↓↓	↓↓/↓/↓↓
	(anti-CTLA4 & anti PD1-L)						
CD8+ (total)	Anti-CTLA4	↑ (clonal expansion in LN)	↑	↑	↑/↑/↑		NA
CD8+ (total)	anti-PD1 (anti-PD1-L)	↑ (effect in the TME)	↑ (↑)	↑	↑/↑/↑		NA
					(↑/↑↑/↑)		
CD8+ (total)	anti-CTLA4 & anti-PD1	↑↑	↑↑	↑↑	↑↑/↑/↑↑		NA
	(anti PD1-L)		(↑↑)		(↑↑/↑↑/↑↑)		
CD8+ PD1+CTLA4−	anti-CTLA4	no effect *	no effect		NA		NA
CD8+ PD1+CTLA4−	anti-PD1	↑	↑		NA		NA
CD8+ PD1+CTLA4−	anti-CTLA4 & anti-PD1	↑ * (same effect as anti-PD1 alone)	↑		NA		NA
CD8+ PD1+CTLA4+	anti-CTLA4	↑	↑		NA		NA
CD8+ PD1+CTLA4+	anti-PD1	↑	↑		NA		NA
CD8+ PD1+CTLA4+	anti-CTLA4 + anti-PD1	↑↑	↑↑		NA		NA

* No CTLA4 expressed on T- cell surface, zero treatment effect of anti-CTLA4 therapy expected. ** Experimental data include all Tregs, but our model takes into account iTregs only. TILs: Tumour Infiltrating Lymphocytes.

**Table 3:** Results from the logical model simulations of T cell response following different types of immune checkpoint inhibitors (ICI) therapy in the lymph nodes and in the tumour microenvironment. (↑ corresponds to upregulation and ↓ corresponds to downregulation of phenotype after ICI treatment. The intensity of the response is such that: ↑↑↑>↑↑>↑). The last column shows the reported efficacy of treatments in a clinical study [5].

Therapy	LN: Proliferation	LN: Th1_CTL/Treg Ratio	TME: Proliferation	TME: Th1_CTL/Treg Ratio	Clinical Outcome: (Patients Survival) [5]
anti-CTLA4	↑	↑	no effect	no effect	↑
anti-PD1	no effect	no effect	↑	↑	↑↑
anti-PD1+anti-CTLA4	↑	↑	↑	↑	↑↑↑

LN: lymph node; CTL: cytotoxic T lymphocyte; Treg: T regulatory.

## Supplementary Materials

The following are available online at https://www.mdpi.com/2072-6694/12/12/3600/s1, Figure S1: MaBoSS simulations of transcription factors FOXP3, FOXO1, and Tbet. Exploration of the role of the transcription factors downstream of the cytokines (left panel) and of the intermediates, STAT1 and STAT3 (right panel), in the activation of the transcription factors with cells treated with anti-PD1 and anti-CTLA4. Results of the simulations are represented as pie charts and inform on the probabilities of each phenotype. PD1/CTLA4 refers to the absence of the two inhibitors for the 6 cases: Tbet ON and OFF, FOXP3 ON and OFF, and FOXO1 ON and OFF. Figure S2: Differential gene expression results were used as initial conditions for our model simulations in both responders and non-responders conditions. For non-responders, we set as initial conditions: TIM3_L, LAG3_L, TIGIT_L, PD1_L, TNFRs_L, ICOS_L; and for responders: LAG3_L, TIGIT_L, TIGIT, PD1_L, PD1, TNFRs, ICOS, IFNG, IL12R, STAT4, Tbet. Table S1: Genes (ligands, receptors, and transcription factors only) of the corresponding proteins presented in our Boolean model.
