# Supplementary material for: Modelling of Immune Checkpoint Network Explains Synergistic Effects of Combined Immune Checkpoint Inhibitor Therapy and the Impact of Cytokines in Patient Response
Source: Cancers (Basel). 2020 Dec 2;12(12):3600. doi: 10.3390/cancers12123600 (PMC7761568; doi:10.3390/cancers12123600)
Supplement: Supplementary file 1 [file cancers-12-03600-s001.zip › cancers-999125-supple-R3/Suppl/FigS2.pdf]

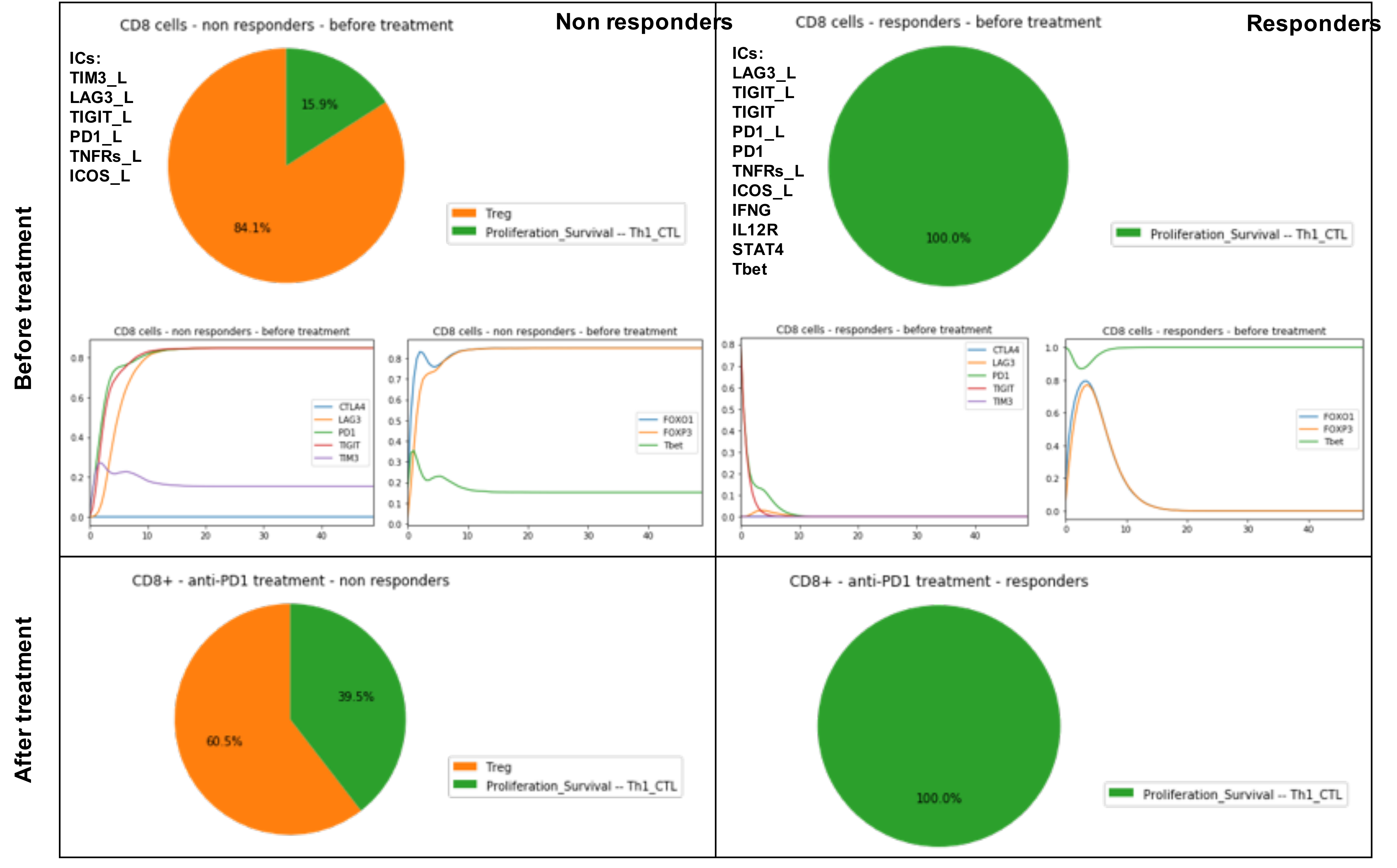

Non responders
Responders
Before treatment
After treatment
CD8 cells - non responders - before treatment
ICs:
TIM3_L
LAG3_L
TIGIT_L
PD1_L
TNFRs_L
ICOS_L
15.9%
84.1%
Treg
Proliferation_Survival -- Th1_CTL
CD8 cells - responders - before treatment
ICs:
LAG3_L
TIGIT_L
TIGIT
PD1_L
PD1
TNFRs_L
ICOS_L
IFNG
IL12R
STAT4
Tbet
100.0%
Proliferation_Survival -- Th1_CTL
CD8 cells - non responders - before treatment
CTLA4
LAG3
PD1
TIGIT
TIM3
CD8 cells - non responders - before treatment
FOXO1
FOXP3
Tbet
CD8 cells - responders - before treatment
CTLA4
LAG3
PD1
TIGIT
TIM3
CD8 cells - responders - before treatment
FOXO1
FOXP3
Tbet
CD8+ - anti-PD1 treatment - non responders
39.5%
60.5%
Treg
Proliferation_Survival -- Th1_CTL
CD8+ - anti-PD1 treatment - responders
100.0%
Proliferation_Survival -- Th1_CTL
