# Supplementary material for: Modelling of Immune Checkpoint Network Explains Synergistic Effects of Combined Immune Checkpoint Inhibitor Therapy and the Impact of Cytokines in Patient Response
Source: Cancers (Basel). 2020 Dec 2;12(12):3600. doi: 10.3390/cancers12123600 (PMC7761568; doi:10.3390/cancers12123600)
Supplement: Supplementary file 1 [file cancers-12-03600-s001.zip › cancers-999125-supple-R3/Suppl/Supp_Mat_model_description.docx]

Supplementary Materials: Modelling of immune checkpoint network explains synergistic effects of combined immune checkpoint inhibitor therapy and the impact of cytokines in patient response

Maria Kondratova *, Emmanuel Barillot, Andrei Zinovyev and Laurence Calzone *

**TCR-signalling model description**

[1. TCR model detailed description**…………………………………………………………………………..**1](#_Toc51839534)

[2. Model annotations………………………………………………………………………………………….](#_Toc51839535)3

[3. References…………………………………………………………………………………………….…….](#_Toc51839536)28

4. The GitHub repository……………………………………………………………………………………30

**1. TCR model detailed description**

The full activation of T-cells can be divided into three parts, following the process of adaptive immune response (Figure 1B of main article): (1) activation of T-cell Receptors (TCR); (2) modulation of TCR downstream signalling by co-stimulatory and co-inhibitory immune-checkpoints; and (3) control of T-cell activity by soluble agents (cytokines, prostaglandin, etc.).

The recognition of antigens by T-cells requires a strong and stable cell-cell contact called the immunological synapse [1]. This contact leads to the formation of multiple intracellular molecular complexes, which include MHC-bound antigen molecules from one side and TCR and CD4/CD8 receptors from the other side. CD4 recognizes the antigens of MHCII complexes and CD8 those of MHCI complexes. The antigen recognition by TCR leads to the formation and activation of LAT protein complexes, including, among other proteins, VAV and PLCG. LCK/FYN kinases also participate in this process via phosphorylations of key molecules of TCR complexes. This LAT-signalosome initiates several downstream signalling pathways such as Ca2+/NFAT, MAPKs (p38, JNK, ERK) and NFkB [2]. These pathways acting together induce the expression of the interleukin-2, IL2, which is a key regulator of T-cell proliferation and clonal expansion. Another signalling pathway needed for a complete T-cell activation is the PI3K/AKT pathway, which reinforces the survival of activated T-cells. TCR signalling alone is not able to maintain a stable and long-term PI3K activation but requires a co-stimulation via CD28 pathway. This process recapitulates the first key stage of T-cell activation [2,3].

The presence and combination of the immune checkpoints affect the activity of the four downstream signalling pathways mentioned above: PI3K_AKT, NFkB, MAPKs and Ca_NFAT pathways, whose state will influence the fate of the T-cells. The activating immune checkpoints will tend to maintain the pathway activity whereas the inhibiting immune checkpoints will shut them off. We have included in the model only some of the well-studied checkpoints with known molecular mechanisms of action: CTLA4, PD1, TIGIT, LAG3, and TIM3 as the inhibiting immune checkpoints and CD28, CD226, ICOS and TNFRs (representing a family of TNFR-like checkpoints) as the activating immune checkpoints. We anticipate that the intensity of each of them and their combination will lead to different outcomes but for this first analysis, we will mainly consider null or full activation of each of the checkpoints (see Jupyter notebook for an example of the gradual inhibition of CTLA4 in the presence of the activating checkpoints).

CTLA4 and LAG3 act as competitors of TCR complexes and block the first steps of T-cell activation. CTLA4 competes with CD28 [4] and LAG3 with CD4 protein (LAG3 binds to MHC class II molecules with a much higher affinity than CD4) [5,6]. At the same level of the signal transduction, TIM3 downregulates the activity of FYN and LCK kinases, which are needed for the first steps of TCR activation [5,7].

TCR activation is mainly governed by the phosphorylation of elements of TCR-complex and downstream adaptors: the SHP phosphatases are able to dephosphorylate molecules of TCR complex and block downstream signalling [8], for instance the ability of PD1 to block T-cell activation correlates with the recruitment of SHP-1 and SHP-2 [9]. The role of SHP phosphatases in CTLA4 signalling is still a subject of controversy. If the first publications demonstrated the binding between SHP proteins and CTLA4 [10], latest studies did not seem to confirm this observation [11], thus, for simplicity, we chose not to include it into the model since its role might be context-dependent.

The downstream signalling pathways are also targeted by the checkpoints: LAG3 blocks Ca2+ flux and NFAT nuclear translocation probably via inhibition of PLCG1 tyrosine phosphorylation [12]. TNF receptors, via TRAF proteins, activate NFkB pathway [13] , and ICOS and CD28 activate MAPKs pathways via adaptor proteins GRB2 and VAV [14]. CD28, ICOS, and TNF receptors activate PI3K pathway via binding of PI3K subunits and some still-undefined mechanisms[13–15] [19–21]. Inhibiting checkpoints use different types of phosphatases to block this key pathway. TIGIT recruits SH2-domain containing inositol-5-phosphatases (SHIP1), which control the activity of the PI3K-dependent signalling cascades via dephosphorylation of PI(3,4,5)P3 [16]. CTLA4-mediated inhibition of Akt phosphorylation is dependent on PP2A phosphatase [17] PD1 increases PTEN activity and prevents PI3K activation through inhibition of Casein Kinase 2, which phosphorylates PTEN and, as a result, suppresses PTEN phosphatase activity [18].

The construction of the network of interactions recapitulating these facts has revealed a number of feedback loops that potentially contribute to the regulation of the expression and the functional activity of the immune checkpoints. PI3K pathway, downstream of TCR receptors, upregulates the expression of Tbet through the glycogen synthase kinase-3 (GSK-3), which leads to the inhibition of PD1 expression[19,20]. PI3K pathway blocks the transcription factor FOXP3 expression [21], which regulates the expression of many checkpoints such as CTLA4, ICOS and TNFRSF18 (GIRT) [22]. Note that CTLA4 is also able to upregulate FOXP3 expression [22,23], thus forming a positive feedback loop between these two proteins.

Activation of the PI3K pathway leads to the phosphorylation of FOXO1, causing its exclusion from the nucleus. TIGIT stimulation represses the PI3K/AKT pathway via SHIP-1, resulting in the activation of FOXO1[16]. FOXO1 is one of the transcription factors promoting the expression of PD1 [24] .

In response to the activation of TCR signalling, cells can proliferate and differentiate [25] Currently, immunologists define about ten basic groups of effector T-cells and a lot of subgroups. Among these ten groups, only two or three might be of particular interest in the context of cancer biology [26]: the regulatory T-cell phenotype driven by FOXP3 and partially FOXO1 (referred to as TREG_phenotype in the model), and Th1 (CD4+) or cytotoxic (mostly CD8+ but also CD4+) phenotypes driven by Tbet (referred to as Th1_Cytotoxicity in the model). These three transcription factors can be seen as “molecular bridges” between the primary TCR signalling, and T-cell differentiation.

FOXO1 and FOXP3 transcription factors are considered as biomarkers of regulatory T-cells (Tregs). In fact, the Treg population is defined in the literature as the population of CD4 positive (CD4+) cells which stably express high level of FOXP3 [27]. Regulatory T-cells play immunosuppressive role in the body: they express high level of inhibiting immune checkpoints on their surface and produce immunosuppressive cytokines TGFB and IL10. FOXP3 and FOXO1 transcription factors play a critical role in the regulation of these immunosuppressive phenotypes. Treg population consists of two subpopulations of natural Tregs (nTregs) and inducible Tregs ( iTregs). The first subpopulation will not be described here because nTregs represent a very specific group of T-cells, which develop in the thymus and whose differentiation does not depend of the mechanisms of T-cell activation explained above, whereas iTreg cells develop from mature CD4+ conventional T-cells, and could be influenced by both immune checkpoints and cytokine signalling [27]. For the rest of the study, iTregs will be referred to as Tregs. Treg phenotype was first described for CD4+ cells only. Later, it was shown that CD8+, as well as CD4+, could express FOXP3 in certain conditions [28,29]. Although the total number of CD8+ FOXP3+ cells is small, they could play an important role in tumour microenvironment (TME). Both CD4+ FOXP3+ and CD8+ FOXP3+ cells inhibit tumour rejection and promote tumour growth [30]. As a general observation, high level of FOXP3+ cells in the TME is associated with a bad outcome [31,32]. FOXO1 another key transcription factor of Treg development is especially important for differentiation of iTreg [33,34].

The third transcription factor, Tbet, is known to regulate the development of Th1 cells (CD4+) and cytotoxic CD8+ and CD4+ cells (together with Ca/NFAT signalling). Among all T-cell population, both cytotoxic (CTLs) and Th1 cells have a significant impact in tumour rejection and patients’ survival. CTLs directly recognize tumour cells and kill them via lytic granule exocytosis and apoptosis induction and Th1 cells in tumours interact with antigen presenting cells (mostly macrophages) and maintain the activity of innate immune response against tumours [35]. The two T-cell populations produce a lot of inflammatory cytokines such as interferon gamma, IFNG. The production of inflammatory cytokines by Th1 and CTLs and the expression of cytotoxic agents in CTLs both require Tbet activity. For simplicity reasons, we chose to ignore these feedbacks in this version of the model and study the ON and OFF effects of the cytokines, how their signals integrate to the immune checkpoints and their impact on the cell fate. That way, we can focus on the effect of the tumour microenvironment on T cell activation.

The expression of these transcription factors is regulated by the crosstalks between TCR signalling pathway, immune-checkpoints and cytokines. Thus, in the T-cell activation network, they play a role of molecular “hubs” integrating all three types of signals (Figure 2). The main cytokine pathway upregulating FOXP3 expression is TGFbeta/SMAD signalling [36,37]. IL12 and IFNG upregulate the expression of Tbet via STAT4 and STAT1 [38], IL27 and IFNG upregulate the expression of both Tbet and FOXP3 via STAT1 [39,40], but IL27 can also inhibit FOXP3 expression via STAT3 [41].

2. Model annotations

We provide here a complete table of annotations for all molecular interactions included in the model.

| FOXO1 -> PD1 | PMID:25464856 | FOXO1 binds to and promotes the expression of PD-1 in CD8+ T cells |
| --- | --- | --- |
| FOXO1 -> FOXP3 | PMID:23318581 | Foxo1 target genes support Treg cell development and function. Foxo1 target genes include Foxp3 (upregulation) and Ifng (downregulation). |
|  | PMID:26363058 | Foxo1 inhibition led to a significant reduction in Foxp3 expression in naive T cells cultured under Treg-skewing conditions |
| FOXO1 -\| IFNG | PMID:23135404, PMID:30728325 | Foxo1 is a pivotal regulator of T(reg )cell function, FOXO1 binds IFNG promoter and inhibits cytokine expression. |
| VAV -> PKC_teta | PMID:14764585 | Vav1 Transduces TCR Signals to Ras and ERK via PLC and DAG  Defective TCR-induced DAG Production in Vav1-deficient Cells  Defective phosphorylation of PKCθ and PKD in Vav1–/– thymocytes. |
| VAV -> MAPKs | PMID:14764585 | Vav1 Transduces TCR Signals to Ras and ERK via PLC and DAG  Defective TCR-induced DAG Production in Vav1-deficient Cells  Defective phosphorylation of PKCθ and PKD in Vav1–/– thymocytes. |
| VAV -> PI3K_AKT | PMID:25539813 | Vav1 is the linker molecule that couples the C-terminal proline-rich motif of CD28 to the recruitment and activation of PIP5Kα, which in turn cooperates with Vav1 in regulating actin polymerization and CD28 signaling functions. |
| STAT4 -> Tbet | PMID:16520391 | STAT4 signal induces T-bet in the absence of STAT1.  PMID:12648458  Activated STAT4 can directly induce IFN-γ production and expression of IL-12Rβ2 and T-bet during Th1 differentiation |
| STAT4 -> TIM3 | PMID:22426209 | By using a T-bet or STAT4 siRNA, we found that TIM-3 expression induced by IL-12 (100 ng/ml) was attenuated on CD4+ T cells transfected with either T-bet or STAT4 siRNA. |
| STAT4 -> IFNG | PMID:16520391 | STAT4 signal induces T-bet in the absence of STAT1.  PMID:12648458  Activated STAT4 can directly induce IFN-γ production and expression of IL-12Rβ2 and T-bet during Th1 differentiation |
| LAG3 -\| CD4 | PMID:22437870; PMID:28900677 | The LAG-3 protein (CD223) is expressed on activated T and NK cells 1, 2 and binds to MHC class II molecules with a much higher avidity than CD4 3. On T cells, LAG-3 associates with the TCR/CD3 complex and, like CTLA-4, negatively regulates signal transduction  Lag-3 has been shown to associate with CD3 and crosslinking of Lag-3 together with CD3 inhibits T cell proliferation, cytokine production, and calcium flux |
| LAG3 -\| Ca_NFAT | PMID:9780176; PMID:31907283 | At the biochemical level, LAG-3XL inhibits calcium response to CD3 stimulation.  locking LAG-3 with an antagonist Ab leads to the activation of NFAT |
| MCHII -> CD4 | PMID:22076556, PMID:22076556, PMID:25720354 | MHC class II molecules present antigen peptides to CD4+ T cells |
| AP1 -> PD1 | PMID:22949674 | Activator protein 1 suppresses antitumor T-cell function via the induction of programmed death 1tumor-infiltrating T cells exhibited persistently up-regulated expression of the activator protein 1 (AP-1) subunit c-Fos during tumor progression. |
| AP1 -> TNFRs | PMID:12706838 | Unlike GITR, which is expressed on all naive T cells, 4-1BB is absent from the surface of resting T cells but is induced by antigen receptor stimulation via the extracellular signal-regulated kinase (ERK) and c-Jun N-terminal kinase (JNK) signaling pathways MEK and JNK1 activities are required in activation‐dependent 4‐1BB upregulation AP‐1 and NF‐κB bind to the putative 4‐1BB promoter binding site |
| AP1 -> FOXP3 | PMID:28757603 | In response to signalling via the TCR and co-stimulation pathways, the FOXP3 promoter is bound and activated by transcription factors such as NFAT and AP-1 |
| AP1 -> IL2 | PMID:10221648 | IL2 expression is a key result of CD4 cells activation, it needs NFkB, AP1 and NFAT transcription factors |
| IL27R -> STAT3 | PMID:25614966 | IL-27 is a potent inducer of Tim-3 in naive CD4+ cells  IL-27-induced NFIL3 and T-bet regulates both Tim-3 and IL-10  STAT1/T-bet and STAT3/NFIL3 mediate IL-27-induced Tim-3, and IL-10, expression.  Both TBET and NFIL3 directly bind TIM3 and IL10 promoters |
|  | PMID:17994025 | IL-27 and IL-6 induced T helper type 1 and type 2 cells, as well as T helper cells that produce IL-17, to secrete IL-10. This effect was dependent on the transcription factors STAT1 and STAT3 for IL-27 and on STAT3 for IL-6. |
| IL27R -> STAT1 | PMID:25614966 | IL-27 is a potent inducer of Tim-3 in naive CD4+ cells  IL-27-induced NFIL3 and T-bet regulates both Tim-3 and IL-10  STAT1/T-bet and STAT3/NFIL3 mediate IL-27-induced Tim-3, and IL-10, expression.  Both TBET and NFIL3 directly bind TIM3 and IL10 promoters |
|  | PMID:17994025 | IL-27 and IL-6 induced T helper type 1 and type 2 cells, as well as T helper cells that produce IL-17, to secrete IL-10. This effect was dependent on the transcription factors STAT1 and STAT3 for IL-27 and on STAT3 for IL-6. |
| FOXP3 -> CTLA4 | PMID:31311918 | Foxp3 upregulates gene expression of CTLA4, ICOS, TNFRSF18, Il2RA in Treg |
|  | PMID:16873067 | Chromatin immunoprecipitation (ChIP) experiments confirmed that NFAT1 and FOXP3 could each occupy the Il2, Ctla4, and Cd25 promoters, both in T cells retrovirally transduced with FOXP3 and in “natural” CD4+CD25+ T regulatory cells that had been expanded with IL-2 |
| FOXP3 -> TNFRs | PMID:31311918 | Foxp3 upregulates gene expression of CTLA4, ICOS, TNFRSF18, Il2RA in Treg |
|  | PMID:12522256 | Foxp3 is known to drive the expression of Treg-associated markers such as CD25, CTLA-4, and GITR |
| FOXP3 -> ICOS | PMID:31311918 | Foxp3 upregulates gene expression of CTLA4, ICOS, TNFRSF18, Il2RA in Treg |
| FOXP3 -> TIGIT | PMID:23974203 | ChIP-qPCR, demonstrared inrichment of FOXP3 at the demethylated TIGIT a  loci in human Treg, as well as at other hypomethylated loci (FOXP3,TSDR, CTLA4, ,and IL2Ra,)known to bind to FOXP3 and probably activates it. |
| FOXP3 -\| SMAD7 | PMID:15100250 | TGF-β induces Foxp3 in CD4+CD25− but not CD4+CD25+ T cells  Foxp3 expression allows TGF-β signaling through down-regulation of Smad7 |
| LCK -> CD4 | PMID:23620508; PMID:21127503 | Lck (lymphocyte-specific tyrosine-protein kinase) is a membrane-tethered kinase that phosphorylates tyrosine residues in the ITAMs in the TCR–CD3 complex. Doubly phosphorylated ITAMs are the docking sites for ZAP70 and other TCR signaling-associated proteins. Lck is often associated with the CD4 or CD8 co-receptors, which might potentiate its activity by bringing it into the proximity of the CD3 chains |
| LCK -> CD8 | PMID:23620508; PMID:21127503 | Lck (lymphocyte-specific tyrosine-protein kinase) is a membrane-tethered kinase that phosphorylates tyrosine residues in the ITAMs in the TCR–CD3 complex. Doubly phosphorylated ITAMs are the docking sites for ZAP70 and other TCR signaling-associated proteins. Lck is often associated with the CD4 or CD8 co-receptors, which might potentiate its activity by bringing it into the proximity of the CD3 chains |
| CD8 -> TCR | PMID:3263576 | CD4 and CD8 are cell-surface glycoproteins expressed on mutually exclusive subsets of peripheral T cells. T cells that express CD4 have T-cell antigen receptors that are specific for antigens presented by major histocompatibility complex class II molecules, whereas T cells that express CD8 have receptors specific for antigens presented by MHC class I molecules |
| Antigen-> TCR | PMID:3263576 | CD4 and CD8 are cell-surface glycoproteins expressed on mutually exclusive subsets of peripheral T cells. T cells that express CD4 have T-cell antigen receptors that are specific for antigens presented by major histocompatibility complex class II molecules, whereas T cells that express CD8 have receptors specific for antigens presented by MHC class I molecules |
| SMAD2_3 -> FOXP3 | PMID:19943263 | TGF-beta is a key signaling factor for Foxp3+ Treg development.  It induces production of FOXP3 both on a MRNA and protein level probably via Smad2/3 |
| SMAD2_3 -\| NFIL3 | PMID:31311918 | Treg cells have the lowest Nfil3 expression level among CD4 +T-cell subsets  TGFB signaling downregulates NFIL3 expression via SMAD3  Overexpression of NFIL3 downregulates Foxp3 in Treg cells |
| SOCS1 -\| IFNGR | PMID:17525754 | SOCS1 negatively regulates both TH1- and TH2-cell differentiation in response to IL-12 and IL-4, respectively the expression of SOCS1 is probably most important for the negative regulation of IFNγ, as robust  IFNγ production was observed in SOCS1-deficient T cells even under neutral differentiation conditions |
| TRAFs -> NFkB | PMID:24484736 | GITR recruits signaling adaptors, TNFR associated factors (TRAFs), of which there are six in mammals, TRAF1-6 (reviewed in [26]). TRAFs 2 and 5 are required downstream of GITR for maximal activation of the MAPK and canonical NF-κB pathways and up-regulation of the anti-apoptotic molecule Bcl-xL |
|  | PMID:22017440 | TRAF1 and TRAF2 are required for maximal MAPK and NFjB activation downstream of 4-1BB in T cells, resulting in upregulation of Bcl-xL and Bﬂ-1 and downmodulation of BIM protein |
| TRAFs -> PI3K_AKT | PMID:21289304 | OX40 after ligation by OX40L assembled a signaling complex that contained the adapter TNFR-associated factor 2 as well as PKB and its upstream activator phosphoinositide 3-kinase (PI3K). |
| PD1 -\| CK2 | PMID:23732914 | PD-1 Increases PTEN Phosphatase Activity While Decreasing PTEN Protein Stability by Inhibiting Casein Kinase 2 |
| PD1 -> SHP | PMID: 28280247 | CD28 is strongly preferred over the TCR as a target for dephosphorylation by PD-1-recruited Shp2 phosphatase. |
| GSK3 -\| Tbet | PMID:22437870; PMID:26885856 | GSK-3 siRNA downregulation, or inhibition by small molecules, blocked PD-1 expression, resulting in increased CD8(+) cytotoxic T lymphocyte (CTL) function. Mechanistically, GSK-3 inactivation increased Tbx21 transcription, promoting enhanced T-bet expression and subsequent suppression of Pdcd1 (encodes PD-1) transcription in CD8(+) CTLs. |
| PI3K_AKT -\|GSK3 | PMID:19836308 | CD28 stimulation inhibits GSK3 by increasing inhibitory serine phosphorylation mediated by the phosphatidylinositol 3-kinase (PI3K) pathway, but independently of the guanine nucleotide exchange factor Vav-1 |
| VAV -\|GSK3 | PMID:16905544 | CD28 stimulation inhibits GSK3 by increasing inhibitory serine phosphorylation mediated by the phosphatidylinositol 3-kinase (PI3K) pathway, but independently of the guanine nucleotide exchange factor Vav-1  GSK-3 phosphorylation is partially dependent on Vav family members and that there is a second pathway that is Vav independent. |
| PD1 -\| ICOS | PMID:12517932 | the ICOS costimulatory effect is negligible in the presence of PD-1 engagement |
| TIM3 -\| FYN | PMID:28300768 | In the absence of ligandmediated Tim-3 signaling, Bat3 is bound to Tim-3 and blocks SH2 domain-binding sites in the Tim-3 tail. In this state, Bat3 recruits the catalytically active form of Lck, thereby forming an intracellular molecular complex with Tim-3 that preserves and potentially promotes T cell signaling. Galectin-9 and Ceacam-1 binding to Tim-3 leads to phosphorylation of Y256 and Y263 and release of Bat-3 from the Tim-3 tail, thereby promoting Tim-3-mediated T cell inhibition by allowing binding of SH2 domain-containing Src kinases and subsequent regulation of TCR signaling, Fyn binds to the same region on the Tim-3 tail as Bat3. Fyn has been implicated in the induction of cell anergy (Davidson et al., 2007) and is known to be a key kinase to activate phosphoprotein associated with glycosphingolipid microdomains (PAG), which recruits Csk to suppress Lck function (Salmond et al., 2009; Smida et al., 2007). Because Fyn and Bat3 bind to the same domain in the Tim-3 cytoplasmic tail, it is possible that a switch between Tim-3-Bat3 and Tim-3-Fyn might trigger the switch of Tim-3 function from being permissive to TCR signaling to inhibition of upstream TCR signaling |
| TIM3 -\| LCK | PMID:28300768; PMID:22863785 | In the absence of ligandmediated Tim-3 signaling, Bat3 is bound to Tim-3 and blocks SH2 domain-binding sites in the Tim-3 tail. In this state, Bat3 recruits the catalytically active form of Lck, thereby forming an intracellular molecular complex with Tim-3 that preserves and potentially promotes T cell signaling. Galectin-9 and Ceacam-1 binding to Tim-3 leads to phosphorylation of Y256 and Y263 and release of Bat-3 from the Tim-3 tail, thereby promoting Tim-3-mediated T cell inhibition by allowing binding of SH2 domain-containing Src kinases and subsequent regulation of TCR signaling , Fyn binds to the same region on the Tim-3 tail as Bat3. Fyn has been implicated in the induction of T cell anergy (Davidson et al., 2007) and is known to be a key kinase to activate phosphoprotein associated with glycosphingolipid microdomains (PAG), which recruits Csk to suppress Lck function (Salmond et al., 2009; Smida et al., 2007). Because Fyn and Bat3 bind to the same domain in the Tim-3 cytoplasmic tail, it is possible that a switch between Tim-3-Bat3 and Tim-3-Fyn might trigger the switch of Tim-3 function from being permissive to TCR signaling to inhibition of upstream TCR signaling |
| SOCS3 -\| IL27R | PMID:17525754 | of SOCS3 protein7. IL-12-induced STAT4 activation is inhibited in TH 2 cells that express high levels of SOCS3  SOCS3 suppresses IL-27 effects, as gp130 (a SOCS3-sensitive receptor) is a component of the IL-27 receptor  SOCS3 binds to gp130-related cytokine receptors, including the phosphorylated tyrosine 757 (Tyr757) residue of gp130 and Tyr800 of IL-12 receptor β2 |
| SOCS3 -\| IL12R | PMID:17525754 | of SOCS3 protein7. IL-12-induced STAT4 activation is inhibited in TH 2 cells that express high levels of SOCS3  SOCS3 suppresses IL-27 effects, as gp130 (a SOCS3-sensitive receptor) is a component of the IL-27 receptor  SOCS3 binds to gp130-related cytokine receptors, including the phosphorylated tyrosine 757 (Tyr757) residue of gp130 and Tyr800 of IL-12 receptor β2 |
| CTLA4 -> PP2A | PMID:16227604 | CTLA-4-mediated suppression of Akt phosphorylation is inhibited by the PP2A inhibitor okadaic acid. |
| CTLA4 -\| CD28 | PMID:22437870 | CD80 and CD86 act lic coactivators of T-cells when they interact with CD28 and  inhibit T-cells via interactions with CTLA4 |
| CTLA4 -> FOXP3 | PMID:22910217; PMID:16517699 | CTLA-4 promotes Foxp3 induction and regulatory T cell accumulation in the intestinal lamina propria  Abs to CTLA-4 block the induction of FoxP3 mRNAexpression |
| NFIL3 -\| FOXP3 | PMID:31311918 | Treg cells have the lowest Nfil3 expression level among CD4 +T-cell subsets  TGFB signaling downregulates NFIL3 expression via SMAD3  Overexpression of NFIL3 downregulates Foxp3 in Treg cells  NFIL3 negatively regulates Treg signature genes ICOS, CTLA4, TNSFRSF18, IL2RA via FOXP3 dependent and independent pathways |
| NFIL3 -\| ICOS | PMID:31311918 | Treg cells have the lowest Nfil3 expression level among CD4 +T-cell subsets  TGFB signaling downregulates NFIL3 expression via SMAD3  Overexpression of NFIL3 downregulates Foxp3 in Treg cells  NFIL3 negatively regulates Treg signature genes ICOS, CTLA4, TNSFRSF18, IL2RA via FOXP3 dependent and independent pathways |
| NFIL3 -\| TNFRs | PMID:31311918 | Treg cells have the lowest Nfil3 expression level among CD4 +T-cell subsets  TGFB signaling downregulates NFIL3 expression via SMAD3  Overexpression of NFIL3 downregulates Foxp3 in Treg cells  NFIL3 negatively regulates Treg signature genes ICOS, CTLA4, TNSFRSF18, IL2RA via FOXP3 dependent and independent pathways |
| NFIL3 -\| CTLA4 | PMID:31311918 | Treg cells have the lowest Nfil3 expression level among CD4 +T-cell subsets  TGFB signaling downregulates NFIL3 expression via SMAD3  Overexpression of NFIL3 downregulates Foxp3 in Treg cells  NFIL3 negatively regulates Treg signature genes ICOS, CTLA4, TNSFRSF18, IL2RA via FOXP3 dependent and independent pathways |
| NFIL3 -> IL10 | PMID:25614966 | IL-27 is a potent inducer of Tim-3 in naive CD4+ cells  IL-27-induced NFIL3 and T-bet regulates both Tim-3 and IL-10  STAT1/T-bet and STAT3/NFIL3 mediate IL-27-induced Tim-3, and IL-10, expression.  Both TBET and NFIL3 directly bind TIM3 and IL10 promoters |
| NFIL3 -> TIM3 | PMID:25614966 | IL-27 is a potent inducer of Tim-3 in naive CD4+ cells  IL-27-induced NFIL3 and T-bet regulates both Tim-3 and IL-10  STAT1/T-bet and STAT3/NFIL3 mediate IL-27-induced Tim-3, and IL-10, expression.  Both TBET and NFIL3 directly bind TIM3 and IL10 promoters |
| TGFb -> TGFR | PMID:24132110 | TGFB1, TGFB2, TGFB3 ligands bind to the type 2 TGFβ receptor (TGFBR2), which causes recruitment and phosphorylation of TGFBR1, resulting in downstream signalling activation. |
| NFAT -> PD1 | PMID:27806234; PMID:18802087 | Transcription of PD-1 in T cells after T-activation requires nuclear translocation of NFAT and binding of NFATc1 (NFAT2) to the PDCD1 promoter.15 FOXO1, Notch, and IRF9 also promote PD-1 transcription, whereas T-bet functions as a transcriptional repressor. |
| NFAT -> ICOS | PMID:17986443 | Overexpression of T-bet or GATA-3 alone could enhance, and NFATc2 could further synergize with either of them to increase, icos transcription. Although T-bet acted on the icos promoter, GATA-3 operated via an icos 3'-un-translated region element. Interestingly, NFATc2 was found to bind promiscuously the icos promoter in developing Th0, Th1, and Th2 cells but became selectively associated with T-bet at the promoter and with GATA-3 at the 3'-untranslated region in fully differentiated Th1 and Th2 cells, respectively. |
| NFAT -> Cytotoxicity | PMID: 28894104 | transcription factor NFATc1 controls the cytotoxicity of mouse cytotoxic T lymphocytes. Activation of Nfatc1 -/- cytotoxic T lymphocytes showed a defective cytoskeleton organization and recruitment of cytosolic organelles to immunological synapses. |
| NFAT -> IL2 | PMID: 8054477 | IL2 expression is akey result of CD4 cells activation, it needs NFkB, AP1 and NF-AT transcription factors |
| CK2 -\| PTEN | PMID:23732914 | PD-1 Increases PTEN Phosphatase Activity While Decreasing PTEN Protein Stability by Inhibiting Casein Kinase 2 |
| PI3K_AKT -\| FOXP3 | PMID: 18509048 | TCR signaling and constitutive PI3K/Akt/mTOR activity antagonised Foxp3 induction. |
| PI3K_AKT -\| FOXO1 | PMID:25464856 | TCR activation of PI3K, AKT, and mTOR and markers of anabolic metabolism are poorly sustained in exhausted CD8+ T cell  AKT phosphorylation inhibits the nuclear activity of FoxO transcription factors, namely FoxO1 |
| PI3K_AKT -> Proliferation_Survival | PMID:17128276 | aurora B, along with survivin and mTOR, as a regulator of the G1-S checkpoint in T cells.  treatment with the PI(3)K inhibitors Ly294002 or wortmannin completely suppressed the induction of aurora B and survivin mRNA and protein. |
| PI3K_AKT -> STAT3 | PMID: 29637003 | The STAT3-Y705 phosphorylation, as activated by PI3K in NK1.1-CD4+NKG2D+ cells |
|  | PMID: 22348200 | STAT3 is phosphorylated downstream of PI3K in many cells |
| ICOSLG -> ICOS | PMID:11007762 | ICOSLG (B7RP1) is expressed on PB B cells and monocytes, and on PB monocyte-derived DC.  It is the ligand to the co-stimulatory protein ICOS of T cells. |
| LAG3_L -> LAG3 | PMID:24769443 | LSECtin, a member of the DC-SIGN family of molecules, is another ligand for Lag-3 (Xu et al., 2014). LSECtin is expressed in the liver and also on many tumors (Xu et al., 2014), thus providing a potential mechanism by which Lag-3-expressing CD8+ T cells and NK cells can be regulated in these tissues |
| TIGIT_L -> TIGIT | PMID:27620276 | Although CD155 is considered the dominant ligand for CD226 and TIGIT, CD226 can also interact with CD112 (24), and TIGIT can interact with CD112 and CD113 (25). |
| TGFR -> SMAD2_3 | PMID:11792802, PMID:12809600 | The canonical TGFB signalling pathway involves phosphorylation of the carboxy-terminal serine residue of the internal modulator SMAD proteins, SMAD2 or SMAD3, by the activated receptors. This phosphorylation induces oligomerization of SMAD2 or SMAD3 with SMAD4, which is necessary for nuclear translocation. |
| PLCG -> Ca_NFAT | PMID:1712101; PMID:25456276 | Functional activation of the T-cell antigen receptor induces tyrosine phosphorylation of phospholipase C-gamma 1.  The exception is T-cell receptor activation, which is linked to PLCG1, not PLCG2.  PLCγ enzymes are mainly activated through tyrosine  phosphorylation by receptor and non-receptor kinases. As in all other PLC families, the main signaling output is generation of the second messengers inositol 1,4,5-trisphosphate (IP3, or InsP3) and diacylglycerol (DAG), from phosphatidylinositol 4,5-bisphosphate (PIP2, or PtdIns(4,5)P2). The released IP3 binds to IP3 receptors on the endoplasmic reticulum resulting in Ca2+ release into the cytoplasm. |
| TIGIT_L -\| CD226 | PMID:22285893 | Immunoglobulin receptors that bind nectin and nectin-like proteins include CD226, TIGIT, CRTAM and CD96  CD226 and TIGIT may control CD8 T cell effector function through mechanisms analogous to that of CD28/CTLA-4 interactions with CD80/86. |
| CD226 -> VAV | PMID:26552706 | DNAM-1 (cd226) controls NK cell activation via an ITT-like motif Upon phosphorylation by Src kinases, this motif enabled binding of DNAM-1 to adaptor Grb2, leading to activation of enzymes Vav-1, phosphatidylinositol 3′ kinase, and phospholipase C-γ1. It also promoted activation of kinases Erk and Akt, and calcium fluxes. Although, as reported, DNAM-1 promoted adhesion, this function was signal-independent and insufficient to promote cytotoxicity. DNAM-1 signaling was also required to enhance cytotoxicity |
| TIGIT -\| CD226 | PMID:22285893 | Immunoglobulin receptors that bind nectin and nectin-like proteins include CD226, TIGIT, CRTAM and CD96  CD226 and TIGIT may control CD8 T cell effector function through mechanisms analogous to that of CD28/CTLA-4 interactions with CD80/86. |
| Tbet -\| PD1 | PMID:21623380 | T-bet directly repressed transcription of the gene encoding PD-1 and resulted in lower expression of other inhibitory receptors. |
| Tbet -> TIM3 | PMID:25614966 | IL-27 is a potent inducer of Tim-3 in naive CD4+ cells  IL-27-induced NFIL3 and T-bet regulates both Tim-3 and IL-10  STAT1/T-bet and STAT3/NFIL3 mediate IL-27-induced Tim-3, and IL-10, expression.  Both TBET and NFIL3 directly bind TIM3 and IL10 promoter |
| Tbet -\| LAG3 | PMID:21623380 | T-bet expression using the retroviral overexpression approach in P14 cells increased KLRG-1 expression (Fig. 8c), but repressed Lag-3, CD160, and BTLA along with PD-1 |
| Tbet -> TH1_cytokines | PMID:12121657 | T-bet is a master regulator of Th1 |
| Tbet -> Cytotoxicity | PMID:16034109 | IL-27 directly acts on naive CD8+ T cells in T-bet-dependent and -independent manners and augments generation of CTL with enhanced granzyme B expression. |
| Tbet -> ICOS | PMID:17986443 | Overexpression of T-bet or GATA-3 alone could enhance, and NFATc2 could further synergize with either of them to increase, icos transcription. |
| Tbet -> IFNG | PMID:11752460 | Regulation of IFN-γ production and Th1 development of CD4 T cells is mediated by IFN-γ/Stat1/T-bet  T-bet is rapidly induced by interferon-gamma in lymphoid and myeloid cells. |
| Tbet -> IL10 | PMID:25614966 | IL-27-induced NFIL3 and T-bet regulates both Tim-3 and IL-10  STAT1/T-bet and STAT3/NFIL3 mediate IL-27-induced Tim-3, and IL-10, expression.  Both TBET and NFIL3 directly bind TIM3 and IL10 promoters |
| PP2A -\| PI3K_AKT | PMID:16227604 | CTLA-4 ligation blocks Akt but not PI3K activation.  PD-1 suppression of PI3K/Akt is dependent upon factors binding to the ITSM motif in its cytoplasmic tail.  CTLA-4-mediated suppression of Akt phosphorylation is inhibited by the PP2A inhibitor okadaic acid. |
| IL12 -> IL12R | PMID:15546391; PMID:12948519 | The IL-12 receptor (IL-12R) is composed of two subunits termed β1 and β2, which are structurally related to the type I cytokine receptor superfamily (44–47). The affinity of IL-12 for either subunit alone is low, but coexpression of both β1 and β2 subunits generates human IL-12 high-affinity binding sites. IL-12p40 interacts predominantly with the β1 subunit, whereas p35 interacts largely with the β2 subunit. |
| MAF -> TIGIT | PMID:29899446 | PRDM1 and c-MAF individually and together upregulate PD1, TIM3, TIGIT, PDPN,PDCCR, LAG3 expression |
| MAF -> TIM3 | PMID:29899446 | PRDM1 and c-MAF individually and together upregulate PD1, TIM3, TIGIT, PDPN,PDCCR, LAG3 expression |
| MAF -> PD1 | PMID:29899446 | PRDM1 and c-MAF individually and together upregulate PD1, TIM3, TIGIT, PDPN,PDCCR, LAG3 expression |
| MAF -> LAG3 | PMID:29899446 | PRDM1 and c-MAF individually and together upregulate PD1, TIM3, TIGIT, PDPN,PDCCR, LAG3 expression |
| SHP -\| CD3 | PMID:19290938 | overexpression of SHP‐1 in T cell lines leads to inhibition of TCR‐mediated phosphorylation of the TCR ζ chain, association of ZAP‐70 with the TCR ζ chain, phosphorylation of LAT, and IL‐2 production |
|  | PMID:22641383 | Programmed cell death 1 forms negative costimulatory microclusters that directly inhibit T cell receptor signaling by recruiting phosphatase SHP2 reduction of CD3ζ phosphorylation was seen at TCR microclusters upon PD-1–PD-L1 binding |
| SHP -\| CD28 | PMID:17531298 | IL-10 receptor-associated tyrosine kinase Tyk-2 acts as a constitutive reservoir for SHP-1 in resting T cells, and then tyrosine phosphorylates SHP-1 on IL-10 binding. SHP-1 rapidly binds to CD28 and ICOS costimulatory receptors and dephosphorylates them within minutes. IL-10 activated SHP-1 inhibits tyrosine phosphorylation and PI3-K binding of CD28 and ICOS  IL-10 does not suppress CD28 or ICOS costimulations in SHP-1–silenced T cells |
|  | PMID:28280247 | CD28 is strongly preferred over the TCR as a target for dephosphorylation by PD-1-recruited Shp2 phosphatase. We also show that CD28, but not the TCR, is preferentially dephosphorylated in response to PD-1 activation by PD-L1 in an intact cell system. These results reveal that PD-1 suppresses T cell function primarily by inactivating CD28 signaling |
| SHP -\| ICOS | PMID:17531298 | IL-10 receptor-associated tyrosine kinase Tyk-2 acts as a constitutive reservoir for SHP-1 in resting T cells, and then tyrosine phosphorylates SHP-1 on IL-10 binding. SHP-1 rapidly binds to CD28 and ICOS costimulatory receptors and dephosphorylates them within minutes. IL-10 activated SHP-1 inhibits tyrosine phosphorylation and PI3-K binding of CD28 and ICOS  IL-10 does not suppress CD28 or ICOS costimulations in SHP-1–silenced T cells |
| IFNG -> IFNGR | PMID:17683974 | IFNG dimer binds to receptor contained two IFNGR1 and two IFNGR2 subunits. |
| NFkB -> TNFRs | PMID:12706838 | AP‐1 and NF‐κB bind to the putative 4‐1BB promoter binding site |
|  | PMID:17641042 | NF-κB might contribute to the up-regulation of OX40 gene expression through chromatin remodeling in activated T cells. |
|  | PMID:24484736 | In conventional T cells, GITR is reciprocally regulated by classical nuclear factor κB (NF-κB; RelA most critical, but cRel and p50 are also important) and nuclear factor of activated T cells (NFAT), with NF-κB inducing and NFAT repressing GITR expression downstream of TCR signals  RelA is a critical positive regulator of GITR  expression, although cRel and NFkB1 also play a positive regulatory role. |
| NFkB -> FOXP3 | PMID:28757603 | In response to signalling via the TCR and co-stimulation pathways, the FOXP3 promoter is bound and activated by transcription factors such as NFAT and AP-1  In Treg cell precursors, TCR stimulation triggers the activation of nuclear factor-κB (NF-κB) family members such as REL. These bind to the constitutively ‘open’ CNS3 enhancer of the FOXP3 gene to initiate transcription. |
| NFkB -> IL2 | PMID:10221648 | IL2 expression is akey result of CD4 cells activation, it needs NFkB, AP1 and NF-AT transcription factors |
| NFkB -> TGFb | PMID:29637003 | TGF-β1 expression in regulatory NK1.1-CD4+NKG2D+ T cells dependents on the PI3K-p85a/JNK/AP1, NF-κB and STAT3 pathways. |
| TNFRs_LGs -> TNFRs |  | by definition |
| IL12R -> STAT4 | PMID:11120802; PMID:9558063 | Stat4 is activated in response to IL-12. |
| MAPKs -> AP1 | PMID:11148124 | JNK/AP1 signaling is activated in T-cells downstream of TCR signaling |
| MAPKs -> YY1 | PMID:30428369 | Transcription factor YY1 is shown to serve as master regulator of T cell exhaustion  YY1 recruits Ezh2 histone methyltransferase to co-repress IL-2 cytokine production  Persistent signal 1+2 stimulation via p38MAPK/JNK pathway promotes YY1 transcription |
| MAPKs -> TNFRs | PMID:12706838 | MEK and JNK1 activities are required in activation‐dependent 4‐1BB upregulation  AP‐1 and NF‐κB bind to the putative 4‐1BB promoter binding site |
| MAPKs -> ICOS | PMID:20116985 | ICOS has diverse functions in CD4+ T cell subsets. Following initial TCR engagement and CD28 costimulation, ICOS is upregulated on CD4+ T cells through NFATc2 and ERK signaling. |
| MAPKs -> FOXP3 | PMID:28757603 | In response to signalling via the TCR and co-stimulation pathways, the FOXP3 promoter is bound and activated by transcription factors such as NFAT and AP-1 |
| MAPKs -> TGFb | PMID:29637003 | TGF-β1 expression in regulatory NK1.1-CD4+NKG2D+ T cells dependents on the PI3K-p85a/JNK/AP1, NF-κB and STAT3 pathways. |
| TIGIT -> SHIP | PMID:23154388, PMID:30728325 | Recruitment of Grb2 and SHIP1 by the ITT-like motif of TIGIT suppresses granule polarization and cytotoxicity of NK cells |
| TIGIT -> IL10 | PMID:24745333 | TIGIT ligation triggered a 2-fold increase in Il10 gene expression by Treg cells in vitro. |
| IL2 -> Proliferation_Survival | PMID:8462103 | L-2-induced T-cell proliferative response is a key determinant affecting the magnitude of the immune response. |
| PTEN -\| PI3K_AKT | PMID:16982858 | PTEN does not act on PI3K directly, but rather dephosphorylates PIP3 on the 3′ position to regenerate PIP2, thus limiting the amount of available PI3K product  Augmented response of PTENΔT T cells is correlated with enhanced activation of the PI3K pathway |
| YY1 -> PD1 | PMID:30428369 | YY1 binds specifically to the consensus sites present in PD1 and Lag3 promoters  PD1 and Lag3 genes are positively regulated by YY1 |
| YY1 -> LAG3 | PMID:30428369 | YY1 binds specifically to the consensus sites present in PD1 and Lag3 promoters  PD1 and Lag3 genes are positively regulated by YY1 |
| PRDM1 -> PD1 | PMID:29899446 | PRDM1 and c-MAF individually and together upregulate PD1, TIM3, TIGIT, PDPN,PDCCR, LAG3 expression |
| PRDM1 -> TIGIT | PMID:29899446 | PRDM1 and c-MAF individually and together upregulate PD1, TIM3, TIGIT, PDPN,PDCCR, LAG3 expression |
| PRDM1 -> TIM3 | PMID:29899446 | PRDM1 and c-MAF individually and together upregulate PD1, TIM3, TIGIT, PDPN,PDCCR, LAG3 expression |
| PRDM1 -> LAG3 | PMID:29899446 | PRDM1 and c-MAF individually and together upregulate PD1, TIM3, TIGIT, PDPN,PDCCR, LAG3 expression |
| ICOS -> MAPKs | PMID:12039907 | mitogen-activated protein kinase family kinases were activated in different ways by CD28 and H4/ICOS. The strong phosphorylation of p46 c-Jun N-terminal kinase was observed upon CD28 co-stimulation, but was less potently induced by H4/ICOS in murine activated CD4(+) |
| ICOS -> PI3K_AKT | PMID:27559335; PMID:18641334 | Similar to CD28 cross-linking, ICOS ligation can yield the recruitment of p50α (27) and p85α (29)  regulatory subunits of PI3K, in conjunction with recruitment of the p110δ catalytic subunit (27, 29).  However, ICOS ligation of activated CD4+ T cells was demonstrated to enhance production of PIP3 (27, 30) and induce stronger Akt phosphorylation than CD28 cross-linking |
| ICOS -> MAF | PMID:12818161; PMID:19098919 | ICOS regulates IL-21 production through c-Maf in Tfh  c-Maf Expression Is Selectively Impaired in ICOS−/− Effector T Cells |
| ICOS -> IL10 | PMID:12145647; PMID:12207353ICOS is | ICOS costimulation of T cells is required for IL-10 production. |
| ICOS -> IL2 | PMID:12145647; PMID:12207353 | ICOS, enhances proliferation of T cells as well as the production of several cytokines  (IL2 IFNG) and enhances anti-tumor responses of CD8+ T cells. |
| ICOS -> IFNG | PMID:12145647; PMID:12207353 | ICOS, enhances proliferation of T cells as well as the production of several cytokines  (IL2 IFNG) and enhances anti-tumor responses of CD8+ T cells. |
| PKC_teta -> NFkB | PMID:15536066 | Role for protein kinase Ctheta (PKCtheta) in TCR/CD28-mediated signaling through the canonical but not the non-canonical pathway for NF-kappaB activation. |
| Ca_NFAT -> NFAT | PMID:10089876, PMID:7650486, PMID:9973469 | Ca('2+) activates Calmodulin 2 ( Calmodulin )/ Protein phosphatase 3 (Calcineurin ) signal. Activated Calcineurin dephosphorylates Nuclear factor of activated T-cells cytoplasmic calcineurin-dependent 2 ( NF-AT1(NFATC2) ). |
| Ca_NFAT -\| TNFRs | PMID:24484736 | NFAT acts as a negative regulator of GITR expression. |
| Ca_NFAT -> CTLA4 | PMID:17785820 | NFAT1 binds to the promoter of the CTLA-4 gene after stimulation by chromatin immunoprecipitation. The functional requirement of the NFAT site for CTLA-4 transcription was demonstrated by mutations in the NFAT site that abolished the activity of the promoter. Furthermore, inhibitors of NFAT suppressed CTLA-4 gene expression, indicating that NFAT plays a critical role in regulating the induction of the CTLA-4 gene in lymphocytes ather their activation. |
| Ca_NFAT -> PD1 | PMID:18802087 | NFATc1 regulates PD-1 expression upon T cell activation. Mutation of the NFATc1 binding site in PD-1 reporter constructs resulted in a complete loss of promoter activity. |
| Ca_NFAT -> ICOS | PMID: 16880206 | Ectopic expression of NFATc2 or a constitutively active MEK2 amplifies ICOS transcription and transactivates a 288-bp core region of the icos promoter in luciferase reporter assays. |
| Ca_NFAT -> FOXP3 | PMID:28757603 | In response to signalling via the TCR and co-stimulation pathways, the FOXP3 promoter is bound and activated by transcription factors such as NFAT and AP-1 |
| GSK3 -\| NFAT | PMID:19836308; PMID:11406367 | GSK3 inactivates NFATc by phosphorylation-dependent stimulation of NFATc nuclear export |
| IL10-> SHP | PMID:17531298 | IL-10 receptor-associated tyrosine kinase Tyk-2 acts as a constitutive reservoir for SHP-1 in resting T cells, and then tyrosine phosphorylates SHP-1 on IL-10 binding. SHP-1 rapidly binds to CD28 and ICOS costimulatory receptors and dephosphorylates them IL-10 activated SHP-1 inhibits tyrosine phosphorylation and PI3-K binding of CD28 and ICOS  IL-10 does not suppress CD28 or ICOS costimulations in SHP-1–silenced T cells |
| IL10 -> IL10R | PMID:21115385; PMID:10433356 | IL10  IL10  The IL10/IL10R1A ‬interaction changes the cytokine conformation allowing the association of the IL10/IL10RA ‬complex with IL10RB. |
| IL10-> LAG3 | PMID:20385810 | Expression of LAG-3 and on CD8+ T cells was up-regulated by IL-10, IL-6 probably via STAT3 |
|  | PMID:16288283 | Foxp3 and Lag-3 in Stat3–/–mice were considerably reduced |
| LAT -> PLCG | PMID:10811803; PMID:9846483 | The linker for activation of T cells (LAT) is a critical adaptor molecule required for T cell antigen receptor (TCR)-mediated signaling and thymocyte development. Upon T cell activation, LAT becomes highly phosphorylated on tyrosine residues, and Grb2, Gads, and phospholipase C (PLC)-γ1 bind LAT via Src homology-2 domains. In LAT-deficient mutant Jurkat cells, TCR engagement fails to induce ERK activation, Ca2+ flux, and activation of AP-1 and NF-AT. |
| VAV -> LAT | PMID:14764585 | Vav1 Transduces T Cell Receptor Signals to the Activation of the Ras/ERK Pathway via LAT, Sos, and RasGRP1*  pathway. In Vav1-deficient cells there is a failure to form a LAT-Grb2-Sos complex following TCR stimulation, probably because of reduced phosphorylation of key tyrosine residues on LAT. This in turn may contribute to the profound defect in TCR-induced Ras and ERK activation. |
| LCK -> VAV | PMID:9438848 | Tyr174 of Vav is thought to be the site of phosphorylation by Lck that regulates Vav function |
| LCK -> PKC_teta | PMID:10652356; PMID:17544292 | Regulation of Protein Kinase Cθ Function during T Cell Activation by Lck-mediated Tyrosine Phosphorylation |
| TCR -> LAT | PMID:10811803 | The linker for activation of T cells (LAT) is a critical adaptor molecule required for T cell antigen receptor (TCR)-mediated signaling and thymocyte development. Upon T cell activation, LAT becomes highly phosphorylated on tyrosine residues, and Grb2, Gads, and phospholipase C (PLC)-γ1 bind LAT via Src homology-2 domains. In LAT-deficient mutant Jurkat cells, TCR engagement fails to induce ERK activation, Ca2+ flux, and activation of AP-1 and NF-AT. |
| FYN -> TCR | PMID:10648627 | Fyn was able to induce tyrosine phosphorylation of the TCR and recruitment of the ZAP-70 kinase |
| LCK -> TCR | PMID:23620508; PMID:21127503 | Lck (lymphocyte-specific tyrosine-protein kinase) is a membrane-tethered kinase that phosphorylates tyrosine residues in the ITAMs in the TCR–CD3 complex. Doubly phosphorylated ITAMs are the docking sites for ZAP70 and other TCR signaling-associated proteins. Lck is often associated with the CD4 or CD8 co-receptors, which might potentiate its activity by bringing it into the proximity of the CD3 chains |
| CD4 -> LCK | PMID:23620508; PMID:21127503 | Lck is often associated with the CD4 or CD8 co-receptors, which might potentiate its activity by bringing it into the proximity of the CD3 chains |
| CD8 -> LCK | PMID:23620508; PMID:21127503 | Lck is often associated with the CD4 or CD8 co-receptors, which might potentiate its activity by bringing it into the proximity of the CD3 chains |
| PD1_L -> PD1 | PMID:24076050; PMID:12538684 | PDL1 and PDL2 are expressed in DCs.  Both whole mAb and Fab enhanced T cell activation, showing that PD-L1 and PD-L2 function to inhibit T cell activation. |
| IFNGR -> STAT1 | PMID:11752460 | Regulation of IFN-γ production and Th1 development of CD4 T cells is mediated by IFN-γ/Stat1/T-bet |
| MHCI -> CD8 | PMID:3263576 | CD4 and CD8 are cell-surface glycoproteins expressed on mutually exclusive subsets of peripheral T cells. T cells that express CD4 have T-cell antigen receptors that are specific for antigens presented by major histocompatibility complex class II molecules, whereas T cells that express CD8 have receptors specific for antigens presented by MHC class I molecules |
| MHCII -> LAG3 | PMID:22437870; PMID:28900677 | The LAG-3 protein (CD223) is expressed on activated T and NK cellsand binds to MHC class II molecules with a much higher avidity than CD4 |
| STAT3 -> NFIL3 | PMID:25614966 | IL-27-induced NFIL3 and T-bet regulates both Tim-3 and IL-10  STAT1/T-bet and STAT3/NFIL3 mediate IL-27-induced Tim-3, and IL-10, expression.  Both TBET and NFIL3 directly bind TIM3 and IL10 promoters |
| STAT3 -> MAF | PMID:21215659; PMID:21922060- | STAT3 binds to GATA3, BATF and MAF genes in CD4 cells and activates them. |
| STAT3 -> PRDM1 | PMID:23349024 | IL‐27‐induced Egr‐2 expression is dependent on STAT3  Egr‐2 directly binds to the promoter region of *Prdm1* and enhances its activity |
| PRDM1 -> IL10 | PMID:21378976 | Blimp‐1 is required for IL‐10 production and high ICOS expression in CD4^+^CD25^+^Foxp3^+^ Treg cells  Blimp-1-deficient Treg cells failed to substantially increase their expression of ICOS |
| PRDM1 -> ICOS | PMID:21378976 | Blimp‐1 is required for IL‐10 production and high ICOS expression in CD4^+^CD25^+^Foxp3^+^ Treg cells  Blimp-1-deficient Treg cells failed to substantially increase their expression of ICOS |
| STAT3 -> TGFb | PMID:29637003 | TGF-β1 expression in regulatory NK1.1-CD4+NKG2D+ T cells dependents on the PI3K-p85a/JNK/AP1, NF-κB and STAT3 pathways.  STAT3 inhibition suppresses TGF-B1 expression |
| STAT3 -\| PI3K_AKT | PMID:26363058 | IL-10 inhibits PI3K/Akt pathway and enhances Foxo1 via STAT3  IL10–treated iTregs showed enhanced STAT3 phosphorylation after7 d of culture (Supplemental Fig. 4A). This STAT3 phosphory-lation could be inhibited by 50 ng/ml of StatticV, Incultures in which StatticV was added along with IL-10, no enhancement of Foxp3 or CTLA-4 expression was observed compared with cells cultured without IL-10 Foxo1 in-hibition, IL-10 could not enhance the suppressive activity ofiTregs |
| STAT3 -> SOCS3 | PMID:21182076 | IL‐27 induced rapid STAT1 and STAT3 signaling, enhanced STAT1 protein levels, and induced SOCS1 and SOCS3 expression SOCS1 is rapidly induced as an immediate early gene in response to cytokines activating STAT1. Similarly, SOCS3 is a target gene of STAT3 signaling, but STAT1 also contributes to its induction |
| STAT3 -\| FOXP3 | PMID:28757603; PMID:18156621 | STAT3 activation triggered by IL-6 and other inflammatory cytokines (such as IL-21) can also inhibit Foxp3 expression by obstructing the binding of IL-2-activated STAT5 to elements of the Foxp3 gene  IL-27 inhibits the development of regulatory T cells via STAT3. |
| STAT3 -> IL10 | PMID:25187566 | Cytokine-induced IL-10 expression in T cells is Stat3- or Stat5-dependent.  Stat3 and Stat5 differentially recruit to theIL10promoter and the I-SRE in humanT cells. Performing ChIP, wemapped Stat3 and Stat5 recruitment to theIL10proximal pro-moter and I-SRE |
| TIM3_L -> TIM3 | PMID:27192565; PMID:25363763 | Tim-3 ligands include soluble ligands (galectin-9 and HMGB1) and cell surface ligands (Ceacam-1 and Phosphatidyl serine [PtdSer]). |
| CD4 -> TCR | PMID:3263576 | CD4 and CD8 are cell-surface glycoproteins expressed on mutually exclusive subsets of peripheral T cells. T cells that express CD4 have T-cell antigen receptors that are specific for antigens presented by major histocompatibility complex class II molecules, whereas T cells that express CD8 have receptors specific for antigens presented by MHC class I molecules |
| SHIP -\| PI3K_AKT | PMID:22483603 | SH2-domain containing inositol-5-phosphatase (SHIP) de-phosphorylates PI(3,4,5)P3 at the D5 position of the inositol ring to create PI(3,4)P2. |
| SMAD7 -\| SMAD2_3 | PMID:15100250 | TGF-β induces Foxp3 in CD4+CD25− but not CD4+CD25+ T cells  Foxp3 expression allows TGF-β signaling through down-regulation of Smad7 |
| IL10R -> STAT3 | PMID:26363058 | IL-10, but not IL-6, enhances Foxp3 and CTLA-4 expression ofhuman TGF-b–induced Tregs  IL-10 enhances iTreg phenotype and function through STAT3 |
| STAT1 -> FOXP3 | PMID:19124747 | STAT1-acti-vating cytokines IL-27 and IFN-G amplify TGF-B-induced FOXP3 expression. This study shows STAT1 binding elementswithin the proximal part of the human FOXP3 promoter, |
| STAT1 -> Tbet | PMID:25885435 | IFNB signal increase TIM3 protein lecel, probably via STAT1 |
| STAT1 -> SOCS1 | PMID:21182076 | IL‐27 induced rapid STAT1 and STAT3 signaling, enhanced STAT1 protein levels, and induced SOCS1 and SOCS3 expression SOCS1 is rapidly induced as an immediate early gene in response to cytokines activating STAT1. Similarly, SOCS3 is a target gene of STAT3 signaling, but STAT1 also contributes to its induction |
| STAT1 -> SOCS3 | PMID:21182076 | IL‐27 induced rapid STAT1 and STAT3 signaling, enhanced STAT1 protein levels, and induced SOCS1 and SOCS3 expression SOCS1 is rapidly induced as an immediate early gene in response to cytokines activating STAT1. Similarly, SOCS3 is a target gene of STAT3 signaling, but STAT1 also contributes to its induction |
| CD80_86 -> CTLA4 | PMID:22437870 | CD80 and CD86 act as coactivators of T-cells when they interact with CD28 and  inhibit T-cells via interactions with CTLA4 |
| CD80_86 -> CD28 | PMID:22437870 | CD80 and CD86 act as coactivators of T-cells when they interact with CD28 and  inhibit T-cells via interactions with CTLA4 |
| TNFRs -> TRAFs | PMID:23758787 | The tumor necrosis factor receptor (TNF-R)-associated factor (TRAF) family of intracellular proteins were originally identified as signaling adaptors that bind directly to the cytoplasmic regions of receptors of the TNF-R superfamily [1-3]. There are six known members of the TRAF family (TRAF1 to 6) in mammals. Although a novel protein was named TRAF7 [4], this claim is controversial as the protein does not have the TRAF homology domain that defines the TRAF family |
| TNFRs -\| FOXP3 | PMID:17641007; PMID:17575071 | OX40 inhibits TGFB-mediated Foxp3 induction OX40/OX40L interactions oppose CD28 and IL-2R signals that  promote Foxp3 expression. |
| CD226 -\| IL10 | PMID:26942885 | CD226 blockage by anti-CD226 blocking mAb LeoA1  anti-CD226 mAb LeoA1 inhibited the mRNA expression of IFN-γ and IL-17 while elevated the mRNA level of IL-10 |
| IL27 -> IL27R | PMID:23306374 | IL-27, a heterodimeric cytokine, is composed of a protein related to IL-12p40, encoded by the Epstein–Barr virus (EBV)-induced gene 3 (EBI3), and a unique IL-12p35-like protein, IL-27p28  The IL-27 receptor (IL-27R) is a heterodimer composed of the ligand-binding IL-27Rα (WSX-1, TCCR) protein and the signal-transducing gp130 chain. Coexpression of both receptor subunits is required for signal transduction. |
| CD28 -> PI3K_AKT | PMID:12670393 | CD28 can bind and activate the lipid kinase phosphatidylinositol 3-kinase (PI-3K) |

3. References

[1] M.L. Dustin, The Cellular Context of T Cell Signaling, Immunity. 30 (2009) 482–492. doi:10.1016/j.immuni.2009.03.010.

[2] G. Gaud, R. Lesourne, P.E. Love, Tcell_TCR-sig-regulatory_NatRevImmu2018, Nat. Rev. Immunol. (2018) 1–13. doi:10.1038/s41577-018-0020-8.

[3] A.H. Courtney, W.L. Lo, A. Weiss, TCR Signaling: Mechanisms of Initiation and Propagation, Trends Biochem. Sci. 43 (2018) 108–123. doi:10.1016/j.tibs.2017.11.008.

[4] D.M. Pardoll, The blockade of immune checkpoints in cancer immunotherapy, Nat. Rev. Cancer. 12 (2012) 252–264. doi:10.1038/nrc3239.

[5] A.C. Anderson, N. Joller, V.K. Kuchroo, L1 Anderson, A. C., Joller, N. and Kuchroo, V. K. (2016) Lag-3, Tim-3, and TIGIT: Co-inhibitory Receptors with Specialized Functions in Immune Regulation. Immunity, NIH Public Access 44, 989–1004.ag-3, Tim-3, and TIGIT: Co-inhibitory Receptors with Specia, Immunity. 44 (2016) 989–1004. doi:10.1016/j.immuni.2016.05.001.

[6] E. Di Carlo, P. Cappello, C. Sorrentino, T. D’Antuono, A. Pellicciotta, M. Giovarelli, G. Forni, P. Musiani, F. Triebel, Immunological mechanisms elicited at the tumour site by lymphocyte activation gene-3 (LAG-3) versus IL-12: sharing a common Th1 anti-tumour immune pathway., J. Pathol. 205 (2005) 82–91. doi:10.1002/path.1679.

[7] M. Rangachari, C. Zhu, K. Sakuishi, S. Xiao, J. Karman, A. Chen, M. Angin, A. Wakeham, E.A. Greenfield, R.A. Sobel, H. Okada, P.J. McKinnon, T.W. Mak, M.M. Addo, A.C. Anderson, V.K. Kuchroo, Bat3 promotes T cell responses and autoimmunity by repressing Tim-3–mediated cell death and exhaustion., Nat. Med. 18 (2012) 1394–400. doi:10.1038/nm.2871.

[8] S.M. Stanford, N. Rapini, N. Bottini, Regulation of TCR signalling by tyrosine phosphatases: From immune homeostasis to autoimmunity, Immunology. 137 (2012) 1–19. doi:10.1111/j.1365-2567.2012.03591.x.

[9] J.M. Chemnitz, R. V. Parry, K.E. Nichols, C.H. June, J.L. Riley, SHP-1 and SHP-2 Associate with Immunoreceptor Tyrosine-Based Switch Motif of Programmed Death 1 upon Primary Human T Cell Stimulation, but Only Receptor Ligation Prevents T Cell Activation, J. Immunol. 173 (2004) 945–954. doi:10.4049/jimmunol.173.2.945.

[10] L.E.M. Marengère, P. Waterhouse, G.S. Duncan, H.W. Mittrücker, G.S. Feng, T.W. Mak, Regulation of T cell receptor signaling by tyrosine phosphatase SYP association with CTLA-4, Science (80-. ). (1996). doi:10.1126/science.272.5265.1170.

[11] E. Hui, J. Cheung, J. Zhu, X. Su, M.J. Taylor, H.A. Wallweber, D.K. Sasmal, J. Huang, J.M. Kim, I. Mellman, R.D. Vale, T cell costimulatory receptor CD28 is a primary target for PD-1-mediated inhibition, Science (80-. ). (2017). doi:10.1126/science.aaf1292.

[12] M. Angin, C. Brignone, F. Triebel, A LAG-3–Specific Agonist Antibody for the Treatment of T Cell–Induced Autoimmune Diseases, J. Immunol. 204 (2020) 810–818. doi:10.4049/jimmunol.1900823.

[13] L.K. Ward-Kavanagh, W.W. Lin, J.R. Šedý, C.F. Ware, The TNF Receptor Superfamily in Co-stimulating and Co-inhibitory Responses., Immunity. 44 (2016) 1005–19. doi:10.1016/j.immuni.2016.04.019.

[14] Y. Arimura, H. Kato, U. Dianzani, T. Okamoto, S. Kamekura, D. Buonfiglio, T. Miyoshi-Akiyama, T. Uchiyama, J. Yagi, A co-stimulatory molecule on activated T cells, H4/ICOS, delivers specific signals in T(h) cells and regulates their responses., Int. Immunol. 14 (2002) 555–66. doi:10.1093/intimm/dxf022.

[15] Y. Zhu, S. Yao, B.P. Iliopoulou, X. Han, M.M. Augustine, H. Xu, R.T. Phennicie, S.J. Flies, M. Broadwater, W. Ruff, J.M. Taube, L. Zheng, L. Luo, G. Zhu, J. Chen, L. Chen, B7-H5 costimulates human T cells via CD28H., Nat. Commun. 4 (2013) 2043. doi:10.1038/ncomms3043.

[16] L.E. Lucca, P.-P. Axisa, E.R. Singer, N.M. Nolan, M. Dominguez-Villar, D.A. Hafler, TIGIT signaling restores suppressor function of Th1 Tregs., JCI Insight. 4 (2019). doi:10.1172/jci.insight.124427.

[17] R. V. Parry, J.M. Chemnitz, K.A. Frauwirth, A.R. Lanfranco, I. Braunstein, S. V. Kobayashi, P.S. Linsley, C.B. Thompson, J.L. Riley, CTLA-4 and PD-1 Receptors Inhibit T-Cell Activation by Distinct Mechanisms, Mol. Cell. Biol. 25 (2005) 9543–9553. doi:10.1128/mcb.25.21.9543-9553.2005.

[18] N. Patsoukis, L. Li, D. Sari, V. Petkova, V.A. Boussiotis, PD-1 Increases PTEN Phosphatase Activity While Decreasing PTEN Protein Stability by Inhibiting Casein Kinase 2, Mol. Cell. Biol. (2013). doi:10.1128/mcb.00319-13.

[19] J.E. Wood, H. Schneider, C.E. Rudd, TcR and TcR-CD28 engagement of protein kinase B (PKB/AKT) and glycogen synthase kinase-3 (GSK-3) operates independently of guanine nucleotide exchange factor VAV-1., J. Biol. Chem. 281 (2006) 32385–94. doi:10.1074/jbc.M604878200.

[20] A. Taylor, J.A. Harker, K. Chanthong, P.G. Stevenson, E.I. Zuniga, C.E. Rudd, Glycogen Synthase Kinase 3 Inactivation Drives T-bet-Mediated Downregulation of Co-receptor PD-1 to Enhance CD8(+) Cytolytic T Cell Responses., Immunity. 44 (2016) 274–86. doi:10.1016/j.immuni.2016.01.018.

[21] S. Sauer, L. Bruno, A. Hertweck, D. Finlay, M. Leleu, M. Spivakov, Z.A. Knight, B.S. Cobb, D. Cantrell, E. O’Connor, K.M. Shokat, A.G. Fisher, M. Merkenschlager, T cell receptor signaling controls Foxp3 expression via PI3K, Akt, and mTOR., Proc. Natl. Acad. Sci. U. S. A. 105 (2008) 7797–802. doi:10.1073/pnas.0800928105.

[22] H.S. Kim, H. Sohn, S.W. Jang, G.R. Lee, The transcription factor NFIL3 controls regulatory T-cell function and stability., Exp. Mol. Med. 51 (2019) 80. doi:10.1038/s12276-019-0280-9.

[23] M.J. Barnes, T. Griseri, A.M.F. Johnson, W. Young, F. Powrie, A. Izcue, CTLA-4 promotes Foxp3 induction and regulatory T cell accumulation in the intestinal lamina propria., Mucosal Immunol. 6 (2013) 324–34. doi:10.1038/mi.2012.75.

[24] V.A. Boussiotis, Molecular and Biochemical Aspects of the PD-1 Checkpoint Pathway., N. Engl. J. Med. 375 (2016) 1767–1778. doi:10.1056/NEJMra1514296.

[25] K. Takase, T. Saito, T cell activation, in: Ryumachi, 1995: pp. 853–861. doi:10.1146/annurev.immunol.021908.132706.

[26] H.J. Kim, H. Cantor, CD4 T-cell subsets and tumor immunity: the helpful and the not-so-helpful, Cancer Immunol. Res. (2014). doi:10.1158/2326-6066.CIR-13-0216.

[27] D.O. Adeegbe, H. Nishikawa, Natural and induced T regulatory cells in cancer, Front. Immunol. 4 (2013). doi:10.3389/fimmu.2013.00190.

[28] J.A. Kapp, R.P. Bucy, CD8+ suppressor T cells resurrected, Hum. Immunol. 69 (2008) 715–720. doi:10.1016/j.humimm.2008.07.018.

[29] S. Wei, I. Kryczek, L. Zou, B. Daniel, P. Cheng, P. Mottram, T. Curiel, A. Lange, W. Zou, Plasmacytoid dendritic cells induce CD8+ regulatory T cells in human ovarian carcinoma, Cancer Res. 65 (2005) 5020–5026. doi:10.1158/0008-5472.CAN-04-4043.

[30] Y. Kiniwa, Y. Miyahara, H.Y. Wang, W. Peng, G. Peng, T.M. Wheeler, T.C. Thompson, L.J. Old, R.F. Wang, CD8+ Foxp3+ regulatory T cells mediate immunosuppression in prostate cancer, Clin. Cancer Res. 13 (2007) 6947–6958. doi:10.1158/1078-0432.CCR-07-0842.

[31] A. Balsari, A. Merlo, P. Casalini, M.L. Carcangiu, C. Malventano, T. Triulzi, S. Menard, E. Tagliabue, FOXP3 expression and overall survival in breast cancer, J. Clin. Oncol. 27 (2009) 1746–1752. doi:10.1200/JCO.2008.17.9036.

[32] D. Wolf, A.M. Wolf, H. Rumpold, H. Fiegl, A.G. Zeimet, E. Muller-Holzner, M. Deibl, G. Gastl, E. Gunsilius, C. Marth, The expression of the regulatory T cell-specific forkhead box transcription factor FoxP3 is associated with poor prognosis in ovarian cancer, Clin. Cancer Res. 11 (2005) 8326–8331. doi:10.1158/1078-0432.CCR-05-1244.

[33] P. Hsu, B. Santner-Nanan, M. Hu, K. Skarratt, C.H. Lee, M. Stormon, M. Wong, S.J. Fuller, R. Nanan, IL-10 Potentiates Differentiation of Human Induced Regulatory T Cells via STAT3 and Foxo1, J. Immunol. 195 (2015) 3665–3674. doi:10.4049/jimmunol.1402898.

[34] Y.M. Kerdiles, E.L. Stone, D.L. Beisner, M.A. McGargill, I.L. Ch’en, C. Stockmann, C.D. Katayama, S.M. Hedrick, Foxo Transcription Factors Control Regulatory T Cell Development and Function, Immunity. 33 (2010) 890–904. doi:10.1016/j.immuni.2010.12.002.

[35] S. Knocke, B. Fleischmann-Mundt, M. Saborowski, M.P. Manns, F. Kühnel, T.C. Wirth, N. Woller, Tailored Tumor Immunogenicity Reveals Regulation of CD4 and CD8 T Cell Responses against Cancer, Cell Rep. (2016). doi:10.1016/j.celrep.2016.10.086.

[36] M.C. Fantini, C. Becker, G. Monteleone, F. Pallone, P.R. Galle, M.F. Neurath, Cutting Edge: TGF-β Induces a Regulatory Phenotype in CD4 + CD25 − T Cells through Foxp3 Induction and Down-Regulation of Smad7 , J. Immunol. (2004). doi:10.4049/jimmunol.172.9.5149.

[37] L. Lu, J. Ma, X. Wang, J. Wang, F. Zhang, J. Yu, G. He, B. Xu, D.D. Brand, D.A. Horwitz, W. Shi, S.G. Zheng, Synergistic effect of TGF-β superfamily members on the induction of Foxp3+ Treg, Eur. J. Immunol. (2010). doi:10.1002/eji.200939618.

[38] Y. Yang, J.C. Ochando, J.S. Bromberg, Y. Ding, Identification of a distant T-bet enhancer responsive to IL-12/Stat4 and IFNγ/Stat1 signals, Blood. 110 (2007) 2494–2500. doi:10.1182/blood-2006-11-058271.

[39] C. Zhu, K. Sakuishi, S. Xiao, Z. Sun, S. Zaghouani, G. Gu, C. Wang, D.J. Tan, C. Wu, M. Rangachari, T. Pertel, H.T. Jin, R. Ahmed, A.C. Anderson, V.K. Kuchroo, An IL-27/NFIL3 signalling axis drives Tim-3 and IL-10 expression and T-cell dysfunction, Nat. Commun. 6 (2015). doi:10.1038/ncomms7072.

[40] N. Ouaked, P.-Y. Mantel, C. Bassin, S. Burgler, K. Siegmund, C.A. Akdis, C.B. Schmidt-Weber, Regulation of the foxp3 Gene by the Th1 Cytokines: The Role of IL-27-Induced STAT1 , J. Immunol. 182 (2009) 1041–1049. doi:10.4049/jimmunol.182.2.1041.

[41] M. Huber, V. Steinwald, A. Guralnik, A. Brüstle, P. Kleemann, C. Rosenplänter, T. Decker, M. Lohoff, IL-27 inhibits the development of regulatory T cells via STAT3, Int. Immunol. (2008). doi:10.1093/intimm/dxm139.

4. The GitHub repository contains:

- supp_mat_model_ICI.bnd: model description in MaBoSS format
- supp_mat_model_ICI.bnd.cfg: configuration file in MaBoSS format with definition of model and simulations parameters
- supp_model_model_ICI.zginml: logical model in GINsim format
- Supp_mat_jupyter_notebook_model_analysis.ipynb: Jupyter notebook of model analysis in python format for reproducibility of the analysis.
- Supp_mat_jupyter_notebook_Exp_validation.ipynb: Jupyter notebook of model validation in python format for reproducibility of the analysis.
