# Supplementary figures and images for: Modelling of Immune Checkpoint Network Explains Synergistic Effects of Combined Immune Checkpoint Inhibitor Therapy and the Impact of Cytokines in Patient Response
Source: Cancers (Basel). 2020 Dec 2;12(12):3600. doi: 10.3390/cancers12123600 (PMC7761568; doi:10.3390/cancers12123600)

## Role of the transcription factors

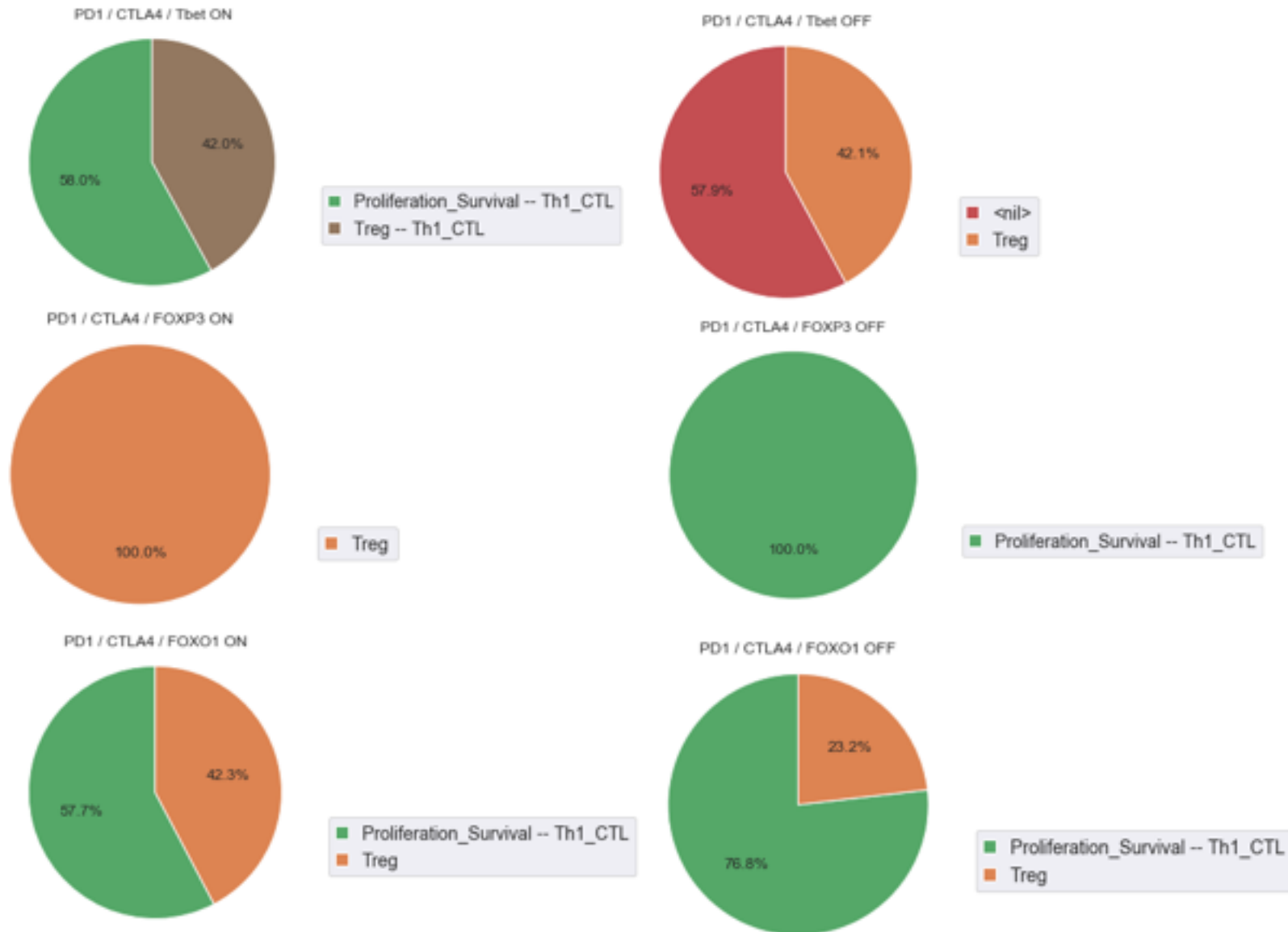

## Role of STAT1 and STAT3

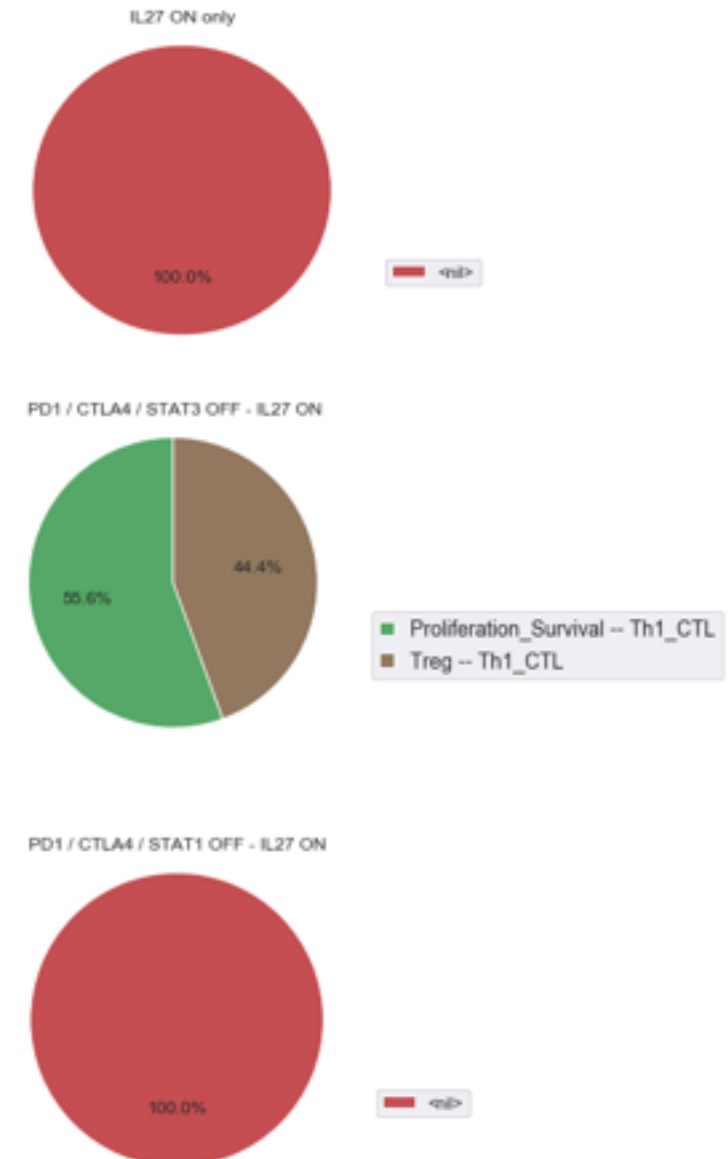

Supplement: Supplementary file 1 [file cancers-12-03600-s001.zip › cancers-999125-supple-R3/Suppl/FigS1.pdf]
